# 14-3-3σ downregulation sensitizes pancreatic cancer to carbon ions by suppressing the homologous recombination repair pathway

**DOI:** 10.18632/aging.205896

**Published:** 2024-06-05

**Authors:** Dandan Wang, Hongtao Luo, Yanliang Chen, Yuhong Ou, Meng Dong, Junru Chen, Ruifeng Liu, Xiaohu Wang, Qiuning Zhang

**Affiliations:** 1 The First School of Clinical Medicine, Lanzhou University, Lanzhou, People’s Republic of China; 2 Institute of Modern Physics, Chinese Academy of Sciences, Lanzhou, People’s Republic of China; 3 Graduate School, University of Chinese Academy of Sciences, Beijing, People’s Republic of China

**Keywords:** carbon ion radiation, 14-3-3σ, pancreatic adenocarcinoma, DNA damage response, homologous recombination repair

## Abstract

This study explored the role of 14-3-3σ in carbon ion-irradiated pancreatic adenocarcinoma (PAAD) cells and xenografts and clarified the underlying mechanism. The clinical significance of 14-3-3σ in patients with PAAD was explored using publicly available databases. 14-3-3σ was silenced or overexpressed and combined with carbon ions to measure cell proliferation, cell cycle, and DNA damage repair. Immunoblotting and immunofluorescence (IF) assays were used to determine the underlying mechanisms of 14-3-3σ toward carbon ion radioresistance. We used the BALB/c mice to evaluate the biological behavior of 14-3-3σ in combination with carbon ions. Bioinformatic analysis revealed that PAAD expressed higher 14-3-3σ than normal pancreatic tissues; its overexpression was related to invasive clinicopathological features and a worse prognosis. Knockdown or overexpression of 14-3-3σ demonstrated that 14-3-3σ promoted the survival of PAAD cells after carbon ion irradiation. And 14-3-3σ was upregulated in PAAD cells during DNA damage (carbon ion irradiation, DNA damaging agent) and promotes cell recovery. We found that 14-3-3σ resulted in carbon ion radioresistance by promoting RPA2 and RAD51 accumulation in the nucleus in PAAD cells, thereby increasing homologous recombination repair (HRR) efficiency. Blocking the HR pathway consistently reduced 14-3-3σ overexpression-induced carbon ion radioresistance in PAAD cells. The enhanced radiosensitivity of 14-3-3σ depletion on carbon ion irradiation was also demonstrated *in vivo*. Altogether, 14-3-3σ functions in tumor progression and can be a potential target for developing biomarkers and treatment strategies for PAAD along with incorporating carbon ion irradiation.

## INTRODUCTION

Abnormal pancreatic gene mutations have been reported to cause uncontrolled growth and division of pancreatic cells and eventually pancreatic cancer (PC) [1]. Pancreatic adenocarcinoma (PAAD) accounts for more than 90% of all PC cases [2]. PAAD is characterized as a devastating disease because of its late diagnosis and extensive metastasis; its 5-year relative survival is only 9% [3, 4], posing a significant public health burden. Therefore, identifying accurate biomarkers and developing comprehensive treatment strategies is imperative for improving PAAD prognosis.

Radiotherapy (RT) is a vital component of PAAD treatment. The current mainstay treatment for nonmetastatic but unresectable locally advanced PC is neoadjuvant therapy consisting of chemotherapy and/or RT [5]. Although X-ray irradiation is the most used irradiation modality and causes damage by producing reactive oxygen species, the highly hypoxic characteristic of PAAD can resist low linear energy transfer (LET) irradiation [6]. Carbon ions, a kind of high LET, exert a strong killing effect on hypoxic cells and thus are effective for PAAD treatment [7]. High-LET exerts its killing effect by generating clustered DNA damage [8, 9]. DNA double-strand breaks (DSBs) are fatal injuries induced by ionizing irradiation, which are repaired largely by error-prone nonhomologous end-joining (NHEJ) and homologous recombination (HR) pathways [10]. NHEJ occurs early (within 30 min after radiation), has high efficiency, and works throughout the cell cycle. In contrast, HR acts mainly in the S and G2 phases [11, 12], its repair takes longer, and its efficiency is only one-third of that of the NHEJ pathway [13].

DNA end resection is an important procedure of the HR pathway [14–16]. In mammals, the cooperation of the MRE11-RAD50-NBS1 (MRN) complex and CTIP forms a 50 to 100 nucleotide 3’-OH single-stranded DNA (ssDNA), then exonuclease 1 (EXO1) and DNA2 process the early intermediate to generate extensive area of ssDNA, following which replication protein A (RPA) coats the 3’-OH ssDNA to remove the secondary structure. Afterward, RAD51 replaces RPA and facilitates the invasion of the DNA double-stranded template to complete the repair process. Targeting DNA damage response (DDR)-associated proteins is significant in the radiosensitization of cancer therapy [17]. Evidence demonstrates that MRE11, CTIP, and Werner (WRN) proteins play major roles in the resection of late and persistent DSBs following irradiation [18]. The DNA damage caused by different LET irradiation activates different DNA repair pathways [19], and the clustered DNA damage caused by high-LET is mainly repaired by HR pathway [20–22]. The characteristics and mechanisms of DNA damage and repair caused by carbon ions are unclear now. Elucidating the underlying molecular mechanism of the tumor reaction to carbon ions can help us understand the carbon ion-specific radiosensitization effect.

The underlying prognostic biomarker and its biological functions in PAAD after carbon ion irradiation were researched in this study. 14-3-3σ (*stratifin*, SFN) is a member of 14-3-3 proteins family in humans that promote cancer progression. In addition, it is an intracellular chaperone in signal transduction in tumorigenesis. 14-3-3σ protects cancer cells from genotoxic agents by mediating proliferation and invasion in gastric cancer [23], anoikis resistance in hepatocellular carcinoma [24], and cell cycle progression in PAAD [25, 26]. In addition, it leads to poor prognosis by regulating breast cancer cells invasion [27]. However, the function 14-3-3σ in PAAD after carbon ion irradiation is still unclear.

We found that 14-3-3σ is upregulated when PAAD cells are treated with carbon ions or other DNA damage. 14-3-3σ silencing enhanced the genomic instability and carbon ion radiosensitivity of PAAD cells *in vitro* and *in vivo*. Altogether, 14-3-3σ depletion potentiates carbon ion-induced DNA damage by hindering the recruitment of RAD51 and RPA2 to the DNA damage sites to repair DSBs. In summary, 14-3-3σ downregulation increases the radiosensitivity of PAAD cells to carbon ions by promoting DNA damage in an HRR impediment manner.

## RESULTS

### 14-3-3σ expression in PAAD and its predictive value for prognosis

**Figure 1:**
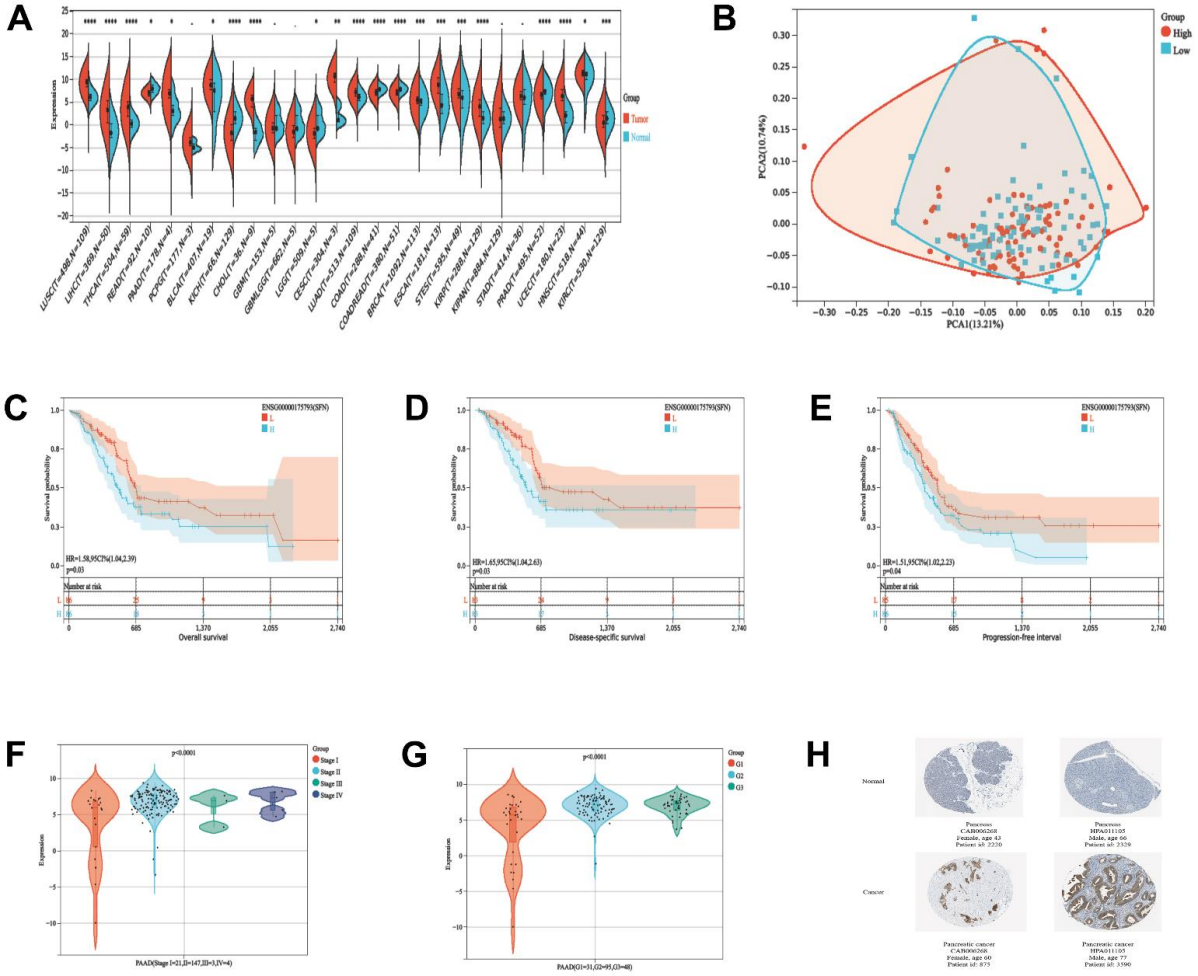
**14-3-3σ expression in PAAD and its predictive value for prognosis.** (**A**) The expression levels of 14-3-3σ mRNA in pan-cancer. (**B**) The principal component analysis (PCA) analysis assessed the grouping situation of patients with different levels of 14-3-3σ expression. The K-M survival curves are plotted to predict the overall survival (OS) (**C**), disease-specific survival (DSS) (**D**), and progression-free interval (PFS) (**E**) of the American Type Culture Collection (TCGA) patients. The expression of 14-3-3σ in PAAD tissues in patients with different Stages (**F**) and Grade (**G**). (**H**) Immunohistochemistry (IHC) results of the expression levels of 14-3-3σ between PAAD and para-carcinoma tissues according to the Human Protein Atlas (HPA) database.

We first analyzed the prognostic importance of 14-3-3σ to elucidate its specific function in PAAD. Patients with PAAD were divided into the low and high 14-3-3σ expression groups through the median values of 14-3-3σ mRNA expression in The Cancer Genome Atlas (TCGA) database. Most cancers express higher 14-3-3σ, including PAAD, than normal tissues in the pan-cancer analysis (Figure 1A). The principal component analysis (PCA) demonstrated acceptable intragroup and intergroup consistencies between the 14-3-3σ-proficient and -deficient groups (Figure 1B). Patients with lower 14-3-3σ expression had more prolonged overall survival (OS) than those with higher expression (*p* = 0.03, hazard ratio [HR]: 1.58, 95% confidence interval [CI]: 1.04–2.39) (Figure 1C), disease-specific survival (DSS) (*p* = 0.03, HR: 1.65, 95% CI: 1.04–2.63) (Figure 1D), and progression-free interval (PFS) (*p* = 0.04, HR: 1.51, 95% CI: 1.02–2.23) (Figure 1E), but not disease-free interval (DFS) (*p* = 0.08, HR: 2.12, 95% CI: 0.90–5.01) (Supplementary Figure 1A). We explored whether the clinicopathologic parameters of patients with PAAD were affected by the 14-3-3σ mRNA levels, and discovered that the expression of 14-3-3σ related to the T stage (Supplementary Figure 1B), pTNM stage (Figure 1F), and grade (Figure 1G); however, its expression remained unaffected by the M stage, N stage, or sex (Supplementary Figure 1C–1E). These results confirmed that the high expression of 14-3-3σ caused poor prognosis in PAAD at the RNA but not at the protein level. Immunohistochemistry (IHC) outcomes demonstrated that patients with PAAD expressed higher 14-3-3σ protein levels than the normal tissues in the Human Protein Atlas (HPA) database (Figure 1H). Univariate logistic regression demonstrated that a higher expression of 14-3-3σ was prone to evolve to an advanced stage (Table 1). In summary, 14-3-3σ expression is increased in PAAD patients, and its high expression can predict malignancy.

**Table 1:** Association between 14-3-4σ expression and clinicopathologic characteristics.

**Clinical characteristics**	**Total (N)**	**The Odds ratio (OR) in 14-3-4σ expression**	**p-value**
Age			
>65 vs. ≤65	181	1.24489 (1.0916-1.420)	0.00109^**^
Gender			
Male vs. Female	181	1.25356 (1.1015,1.427)	0.000614^***^
Stage	178		
II vs. I		1.21241 (1.0502-1.400)	0.00857^**^
III vs. I		1.6197 (1.04517-2.510)	0.031^*^
IV vs. I		1.5861 (1.04179-2.415)	0.0315^*^
T	179		
T2 vs. T1		1.4668 (1.0504-2.048)	0.0245^*^
T3 vs. T1		1.14462 (0.9743-1.345)	0.100
T4 vs. T1		8.572 (0.22378-328.401)	0.248
N			
N1 vs. N0	175	1.18000 (1.024-1.360)	0.0221^*^
M			
M1 vs. M0	85	1.10489 (0.8767-1.393)	0.398

### Knockdown of 14-3-3σ sensitizes PAAD cells

**Figure 2:**
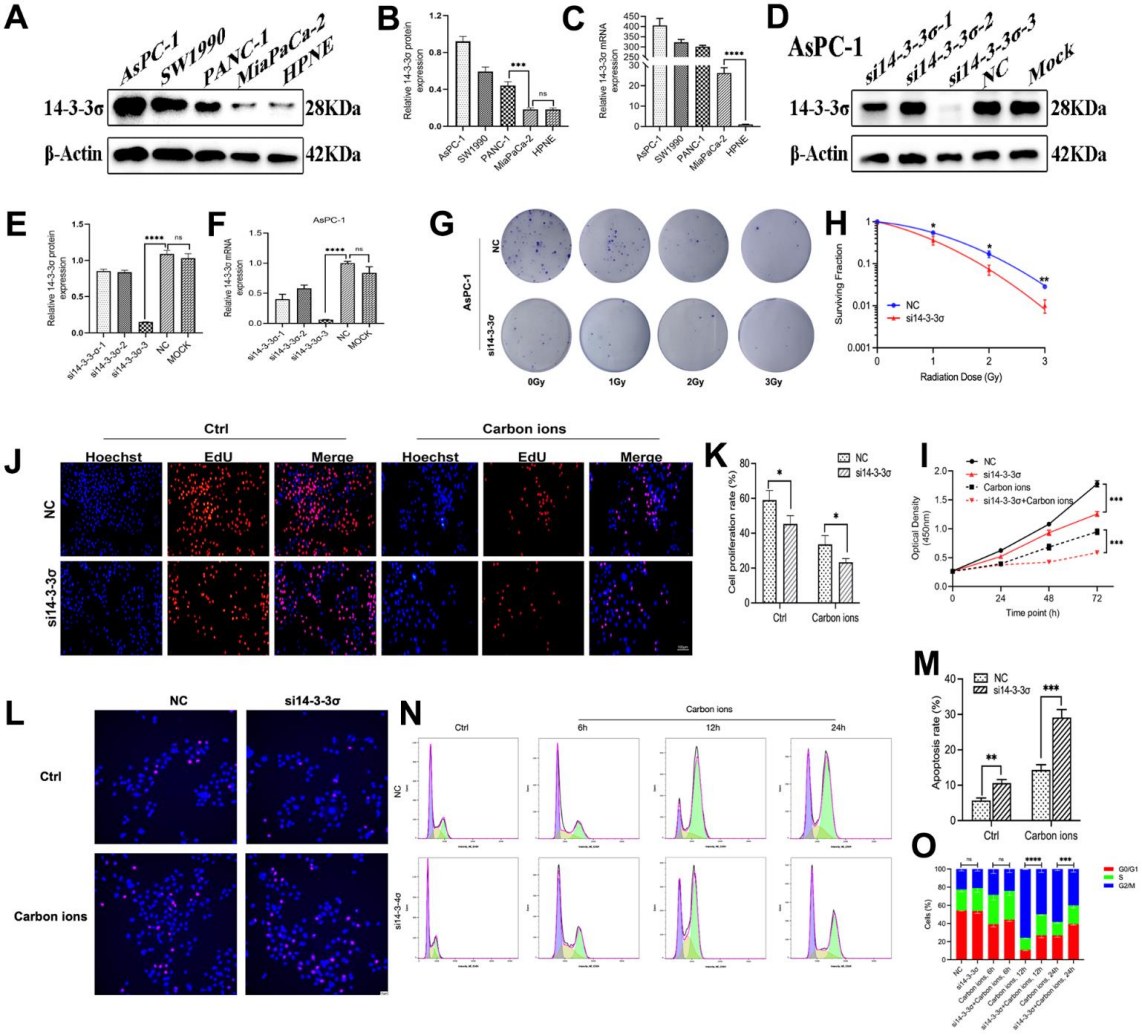
**Knockdown of 14-3-3σ sensitizes PAAD cells.** The level of 14-3-3σ expression in PAAD cell lines (AsPC-1, SW1990, PANC-1, MiaPaCa-2) was detected by immunoblotting (**A**, **B**) and qPCR (**C**). The protein and mRNA levels of 14-3-3σ in AsPC-1 cells with 14-3-3σ-specific small interfering RNA (siRNA) were detected by immunoblotting (**D**, **E**) and qPCR (**F**). (**G**, **H**) Colony formation assays and survival fraction curves of AsPC-1 cells after exposure to the indicated carbon ion irradiation dose (1 Gy, 2 Gy, and 3 Gy). (**I**) Cell proliferation of AsPC-1 cells in different groups after 3Gy carbon ion irradiation was determined by CCK-8 assay at 24 h, 48 h, and 72 h. (**J**, **K**) The proliferation of AsPC-1 cells was detected by EdU assay. Scale bar = 100 μm. (**L**, **M**) The apoptosis rate of AsPC-1 cells after 3 Gy carbon ion irradiation at 24 h in different groups. Scale bar = 10 μm. (**N**, **O**) The cell cycle distribution of AsPC-1 cells after 3 Gy carbon ion irradiation at 6 h, 12 h, and 24 h in different groups. β-Actin was used as a loading control. Data were presented as the mean ± standard deviation (SD) (n=3); *p <0.05, **p <0.01, ***p <0.001, ****p <0.0001, ns: not significant.

**Figure 3:**
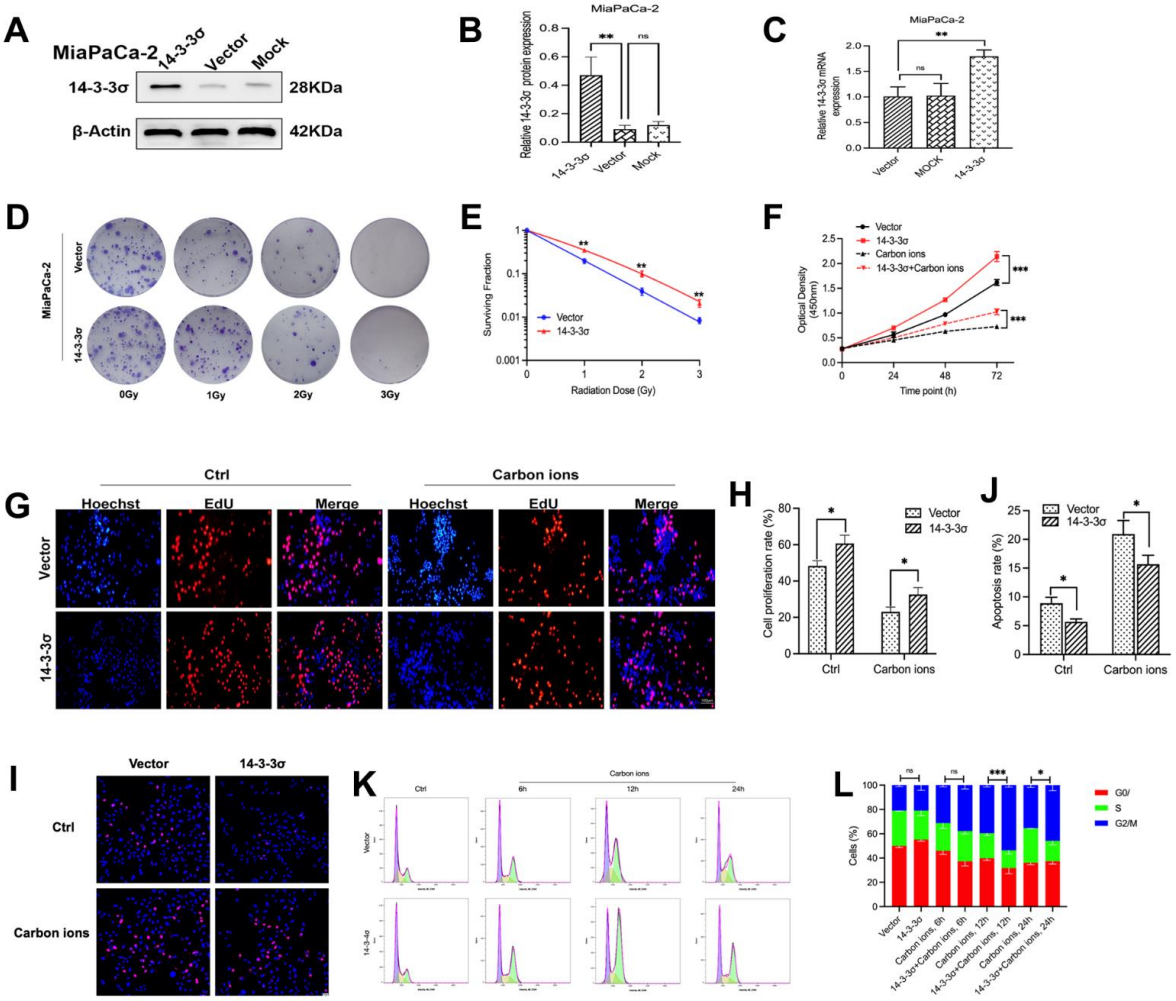
**14-3-3σ overexpression confers carbon ion radioresistance of PAAD cells.** The protein and mRNA levels of 14-3-3σ in AsPC-1 cells with 14-3-3σ overexpression plasmids in MiaPaCa-2 cells were detected by immunoblotting (**A**, **B**) and qPCR. (**C**). (**D**, **E**) Colony formation assays and survival fraction curves of MiaPaCa-2 cells after exposure to the indicated carbon ion irradiation dose (1 Gy, 2 Gy, and 3 Gy). (**F**) Cell proliferation of MiaPaCa-2 in different groups after 3 Gy carbon ion irradiation was determined by CCK-8 assay at 24 h, 48 h, and 72 h. (**G**, **H**) The proliferation of MiaPaCa-2 cells was detected by EdU assay. Scale bar = 100 μm. (**I**, **J**) The apoptosis rate of MiaPaCa-2 cells after 3 Gy carbon ion irradiation at 24 h in different groups. Scale bar = 10 μm. (**K**, **L**) The cell cycle distribution of MiaPaCa-2 cells after 3 Gy carbon ion irradiation at 6 h, 12 h, and 24 h in different groups. β-Actin was used as a loading control. Data were presented as the mean ± standard deviation (SD) (n=3); *p <0.05, **p <0.01, ***p <0.001, ****p <0.0001, ns: not significant.

We next checked the 14-3-3σ expression in four PAAD cell lines (AsPC-1, MiaPaCa-2, PANC-1, and SW1990) and human pancreatic ductal cells (HPNE) using both immunoblotting and quantitative reverse transcriptase-polymerase chain reaction (qRT-PCR) (Figure 2A–2C). The results depicted the PAAD cells abundantly expressed 14-3-3σ. To demonstrate the specific function of 14-3-3σ in PAAD cells in combination with carbon ion radiotherapy, we constructed 14-3-3σ knockdown (AsPC-1) and overexpression (MiaPaCa-2) cells according to their expression in tested PAAD cell lines. We used siRNAs targeting the *14-3-3σ* gene (si14-3-3σ group) or negative control (NC group) to generate 14-3-3σ deficient AsPC-1 cells. Simultaneously, we constructed 14-3-3σ-overexpressing MiaPaCa-2 cells (14-3-3σ group) and empty vector control cells (Vector group) and evaluated the efficiency of knockdown (Figure 2D–2F) and overexpression (Figure 3A–3C) by immunoblotting and qRT-PCR. Consistent with the bioinformatic analysis results that 14-3-3σ supports the proliferation of PAAD, its depletion reduced the colony numbers in AsPC-1 cells after carbon ion irradiation (Figure 2G, 2H and Supplementary Table 1). Notably, 14-3-3σ deficiency markedly increased the radiosensitivity by two-fold in comparison with the parent cells with 2 Gy carbon ion radiation (SF 0.07 vs. 0.17). The cell count kit 8 (CCK8) assay demonstrated that 14-3-3σ deficiency significantly restrained AsPC-1 cell proliferation, with a pronounced inhibition observed following the addition of carbon ions (Figure 2I). We observed the semblable outcomes after the 5-Ethynyl-2′-deoxyuridine (EdU) incorporation assay (Figure 2J, 2K). Afterward, we examined the effect of 14-3-3σ depletion on cell apoptosis and the cell cycle upon carbon ions. After 3 Gy carbon ion radiation, the 14-3-3σ depletion group conferred a significantly higher apoptotic rate (si14-3-3σ group: 29.13%±2.26%; NC group: 14.34%±1.48%) (Figure 2L, 2M). As 14-3-3σ is involved in the eukaryotic cell cycle progression [28, 29]. We next performed flow cytometry to evaluate the effect of 14-3-3σ knockdown on the cell cycle at 6 h, 12 h, and 24 h after carbon ions. The G2/M arrest reached the peak at 12 h after carbon ions. Although unirradiated AsPC-1 cells demonstrated a normal G2/M population upon deficiency of 14-3-3σ, the proportion of cells in the G2 phase was about 75% and 50% for the NC group and si14-3-3σ group at 12 h after 3 Gy of carbon ion radiation, respectively (Figure 2N, 2O), indicating that the 14-3-3σ depletion impaired the G2 phase arrest. Thus, the knockdown of 14-3-3σ impeded PAAD cell vitality synergistically with carbon ion irradiation.

### 14-3-3σ overexpression confers carbon ion radioresistance of PAAD cells

The above data proved that the knockdown of 14-3-3σ restrained PAAD cell vitality and increased carbon ion radiosensitivity. We next wanted to check if increasing the 14-3-3σ expression correspondingly enhanced the radioresistance of PAAD cells. Overexpressed 14-3-3σ significantly enhanced MiaPaCa-2 cells’ tolerance to carbon ion irradiation in the colony-forming assay (Figure 3D, 3E and Supplementary Table 2). Similarly, the overexpression of 14-3-3σ enhanced the proliferation of PAAD cells with or without carbon ions, as revealed by the CCK8 (Figure 3F) and EdU assays (Figure 3G, 3H). The apoptotic ratio in 14-3-3σ-overexpressed MiaPaCa-2 cells (5.69%±0.49%) was significantly lower than that in parental cells (8.92%±1.02%), especially after carbon ion irradiation (15.68%±1.56% vs. 20.93%±2.37%) (Figure 3I, 3J). In 14-3-3σ-overexpression MiaPaCa-2 cells, G2/M arrest was higher than the control cells after carbon ions (Figure 3K, 3L). The above results demonstrated that 14-3-3σ was involved in G2 phase arrest. Since the G2/M phase is a radiosensitive cell cycle phase, the increased expression of 14-3-3σ contributes to the radioresistance of PAAD cells to carbon ions.

### 14-3-3σ is responsive to DNA damage in PAAD cells

**Figure 4:**
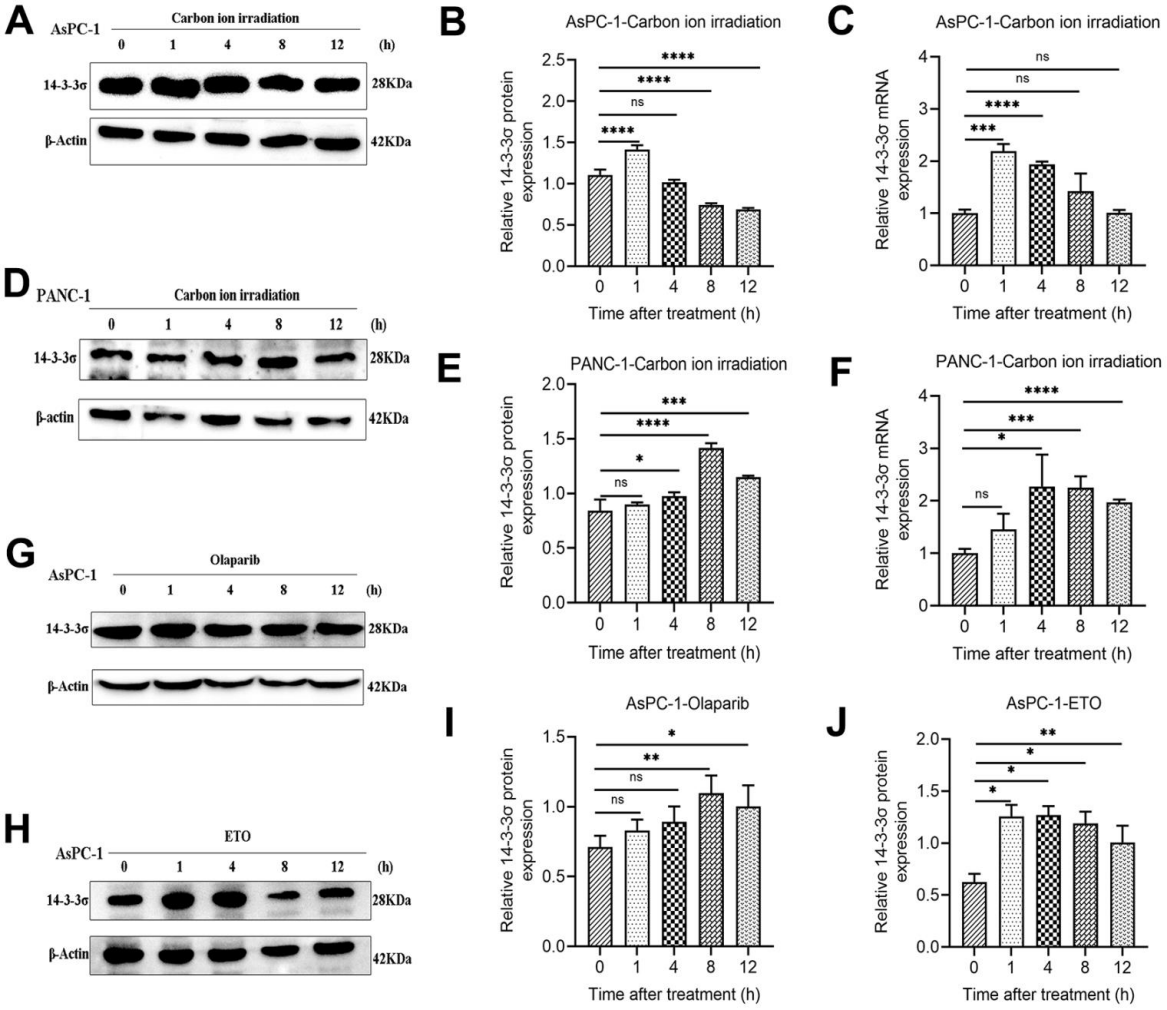
**14-3-3σ is responsive to DNA damage in PAAD cells.** (**A**–**C**) The protein and mRNA levels of 14-3-3σ in AsPC-1 cells after 3 Gy carbon ion irradiation at 1 h, 4 h, 8 h, and 12 h. (**D**–**F**) The protein and mRNA levels of 14-3-3σ in PANC-1 cells after 3 Gy carbon ion irradiation at 1 h, 4 h, 8 h, and 12 h. (**G**–**J**) The protein levels of 14-3-3σ expression in AsPC-1 cells with chemotherapeutics at 1 h, 4 h, 8 h, and 12 h. β-Actin was used as a loading control. Data were presented as the mean ± standard deviation (SD) (n=3); *p <0.05, **p <0.01, ***p <0.001, ****p <0.0001, ns: not significant.

Because 14-3-3σ protects the cells from carbon ion-induced DNA damage, we evaluated 14-3-3σ expression after carbon ion irradiation and other forms of genetic damage at various time points (1, 4, 8, and 12 h) to explore the function 14-3-3σ in DDR in PAAD cells. For tested AsPC-1 and PANC-1 cells, 14-3-3σ expression was significantly enhanced at 1 h and 4 h following exposure to carbon ions and declined at 4 h and 12 h (Figure 4A–4E). The mRNA levels in AsPC-1 and PANC-1 were upregulated following carbon ion irradiation (Figure 4C, 4F). Etoposide (ETO) or olaparib can induce DNA damage and is usually clinically to treat PAAD [30, 31]. We found that AsPC-1 cells expressed a higher level of 14-3-3σ after treatment with these two agents (Figure 4G–4J), demonstrating that 14-3-3σ promoted the recovery of PAAD cells from genetic damage and thus ensured genetic stability.

### 14-3-3σ is involved in the accumulation of DSBs

**Figure 5:**
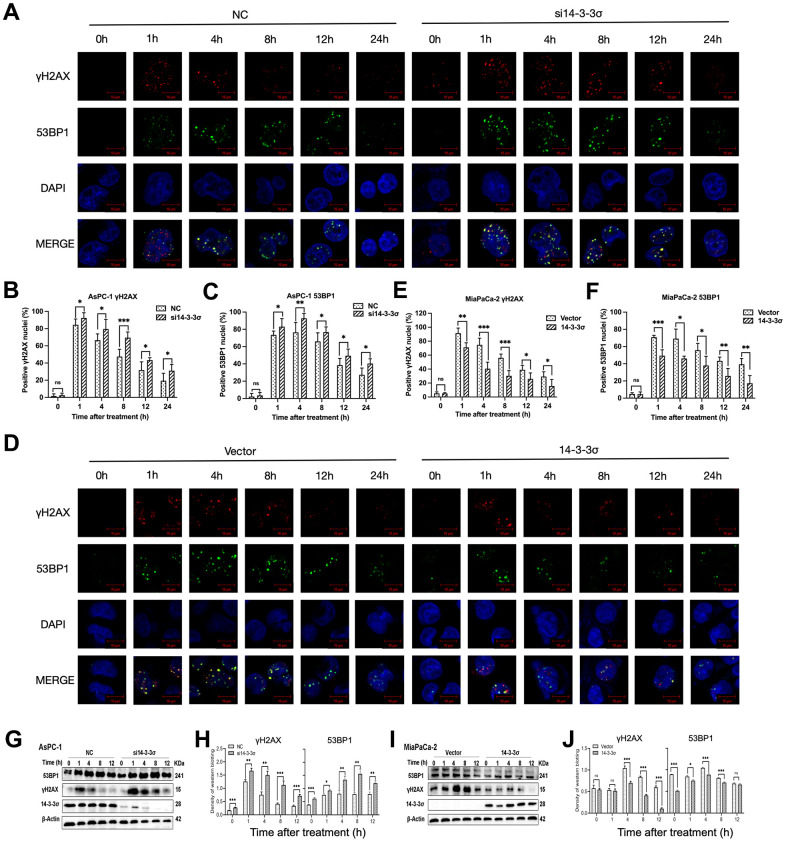
**14-3-3σ involves the accumulation of DSBs.** (**A**) The representative images of the γH2AX and P53 binding protein 1 (53BP1) foci in AsPC-1 cells after 3 Gy carbon ion irradiation in different groups were analyzed by immunofluorescence (IF) staining. (**B**, **C**) Percentage of positive nuclei of γH2AX and 53BP1 in AsPC-1 cells in different groups (5 foci or more per nucleus being considered positive). (**D**) The representative images of the γH2AX and 53BP1 foci in MiaPaCa-2 cells after 3 Gy carbon ion irradiation in different groups were analyzed by IF staining. (**E**, **F**) Percentage of positive nuclei of γH2AX and 53BP1 in MiaPaCa-2 cells in different groups (5 foci or more per nucleus being considered positive). (**G**, **H**) Relative expression of 53BP1 and γH2AX in si14-3-3σ and NC AsPC-1 cells, 0 h, 1 h, 4 h, 8 h, and 12 h post-irradiation. (**I**, **J**) Relative expression of 53BP1 and γH2AX in 14-3-3σ-overexpression and vector MiaPaCa-2 cells, 0 h, 1 h, 4 h, 8 h, and 12 h post-irradiation. For IF, at least 100 cells per condition were analyzed. β-Actin was used as a loading control. Data were presented as the mean ± standard deviation (SD); *p <0.05, **p <0.01, ***p <0.001, ****p <0.0001, ns: not significant. Scale bar = 10 μm.

We next examined the dynamic accumulation of phos-histone (γH2AX) and p53-binding protein 1 (53BP1) following irradiation with carbon ions in these cells at various time points (1, 4, 8, 12, and 24 h) to determine whether the 14-3-3σ-mediated carbon ion radioresistance was due to its effect on DNA repair. γH2AX and 53BP1 are two nuclear proteins most associated with DSB induction. 53BP1 foci are colocalized with γH2AX foci generated in the DSB-containing chromatin domains and therefore could serve as alternative markers for DSB induction and repair [32]. Confocal microscopy demonstrated that the numbers of γH2AX and 53BP1 foci had no difference between the 14-3-3σ-depleted and parental AsPC-1 cells before carbon ion irradiation. The si14-3-3σ group displayed more unresolved γH2AX and 53BP1 foci than the NC group after carbon ion irradiation (Figure 5A–5C), consistent with lower γH2AX and 53BP1 expression and lower DNA damage in 14-3-3σ-overexpressed MiaPaCa-2 cells than in the Vector group (Figure 5D–5F). Moreover, the positive cell rate (five foci or more per nucleus) of γH2AX/53BP1 in the si14-3-3σ and NC groups peaked at 1 h and 4 h, respectively (si14-3-3σ group: γH2AX: 92.49%±6.04%, 53BP1: 92.73%±5.63%; NC group: γH2AX: 84.46%±6.71%, 53BP1: 76.42%±11.48%). The percentage of positive γH2AX and 53BP1 foci cells in the 14-3-3σ knockdown group was retained up to 33.59% and 43.57% of the peak rate at 24 h after carbon ion radiation, respectively, whereas the positive rate of γH2AX and 53BP1 declined to 22.93% and 35.79% in the NC group, respectively, suggesting that 14-3-3σ reduced carbon ion-induced damage with slow repair kinetics. In line with the above results, immunoblotting demonstrated that the depletion of 14-3-3σ caused markedly increased levels of γH2AX and 53BP1 proteins and vice versa (Figure 5G–5J). The above data indicated that the depletion of 14-3-3σ impaired DSB repair capacity, thereby keeping DSBs at high levels.

### Silencing of 14-3-3σ causes the HRR defect after carbon ion radiation

**Figure 6:**
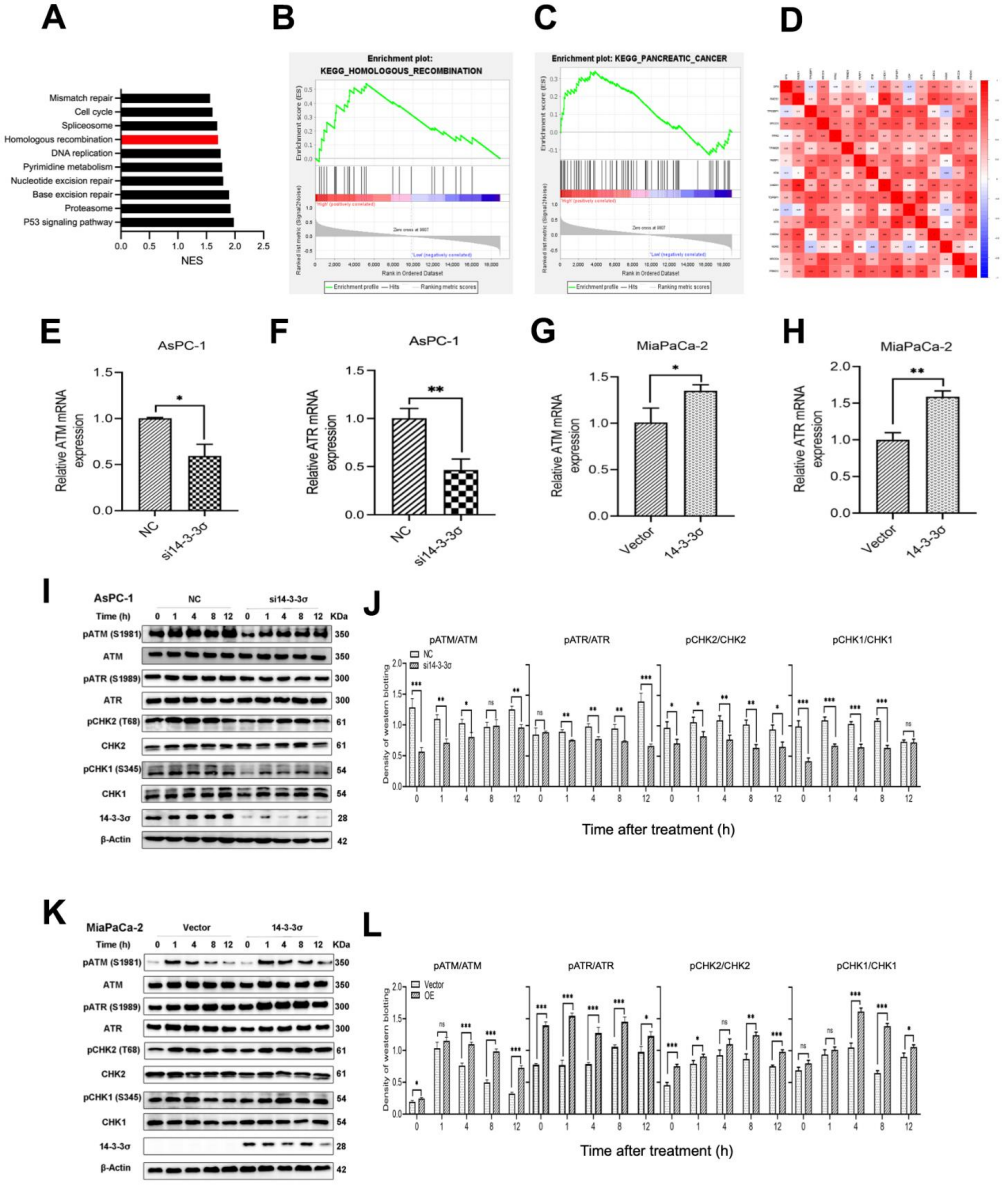
**Silencing of 14-3-3σ causes the HRR defect after carbon ion radiation.** (**A**–**C**) Gene Set Enrichment Analysis (GSEA) for the enriched gene sets in the 14-3-3σ high- and low-expression groups. (**D**) Bioinformatics analysis of the correlation between 14-3-3σ and genes related to homologous recombination (HR) and non-homologous end-joining (NHEJ) pathway. (**E**–**H**) The mRNA expression of ATM and ATR after the silencing or overexpressing of 14-3-3σ in AsPC-1 and MiaPaCa-2 cells. (**I**, **J**) Relative expression of pATM, ATM, pATR, ATR, pCHK2, CHK2, pCHK1, CHK1 in si14-3-3σ and NC AsPC-1 cells, 0 h, 1 h, 4 h, 8 h, and 12 h post-irradiation. (**K**, **L**) Relative expression of pATM, ATM, pATR, ATR, pCHK2, CHK2, pCHK1, CHK1 in 14-3-3σ-overexpression and vector MiaPaCa-2 cells, 0 h, 1 h, 4 h, 8 h, and 12 h post-irradiation. β-Actin was used as a loading control. Data were presented as the mean ± standard deviation (SD) (n=3); *p <0.05, **p <0.01, ***p <0.001, ****p <0.0001, ns: not significant.

DSBs repair ability is critical for the killing effect of irradiation, and HR and NHEJ are the two key repair pathways [33]. We explored the relationship of 14-3-3σ with genes related to the HR and NHEJ pathways, and the results showed that 14-3-3σ was positively correlated with the critical HR genes, including *ATR* (ATM and Rad3-related), *Chk1*, *Chk2, RAD51*, and *H2AX* (all *r>*0.3, *p*-value<0.05) (Figure 6D). The gene set enrichment analysis (GSEA) was conducted then to investigate the specific molecular mechanism related to 14-3-3σ in PAAD, which indicated that the abundant 14-3-3σ was remarkably enriched in the HR pathway (Figure 6A–6C). Evidence has demonstrated that the NHEJ pathway is not essential for restoring clustered DSBs caused by high-LET radiation [34, 35], and carbon ion-induced DSB restoration is more dependent on the HR pathway than the repair following γ-ray or proton irradiation [36]. Thus, we hypothesized that 14-3-3σ released the damage caused by carbon ions in PAAD cells by activating the HRR pathway. Based on this assumption, we conducted qRT-PCR to examine whether 14-3-3σ affected the expression of ATM (ataxia telangiectasia mutated) and ATR, which could perceive DNA damage and activate the HR pathway. Figure 6E–6H shows the attenuated expression of ATM and ATR by deleting 14-3-3σ at the mRNA level. Correspondingly, 14-3-3σ overexpression increased the expression of both ATM and ATR mRNAs. We next extracted the proteins from cells after 3 Gy of carbon ion radiation in each group and checked the influence of 14-3-3σ on the mainstream proteins of the HR signaling pathway. Immunoblotting results demonstrated that the irradiation with carbon ions reduced the activation of phosphorylation of ATM and ATR in 14-3-3σ-depleted AsPC-1 cells (Figure 6I, 6J); in contrast, 14-3-3σ overexpressed MiaPaCa-2 cells displayed increased phosphorylation of ATM and ATR (Figure 6K, 6L). Chk2 and Chk1, downstream factors of ATM and ATR signaling pathways, respectively, were detected subsequently, as displayed in Figure 6K, 6L. We found that the phosphorylation of both Chk2 and Chk1 was attenuated with the silencing of 14-3-3σ and carbon ion irradiation. Conversely, 14-3-3σ overexpression enhanced the phosphorylation of Chk1 and Chk2 with unaffected total proteins (Figure 6K, 6L). Chk2 plays a vital role in the cell cycle course when cells confer DNA damage; its upregulation induced by the overexpression of 14-3-3σ could arrest cells at the G2-M phase and repair the damage induced by carbon ions.

**Figure 7:**
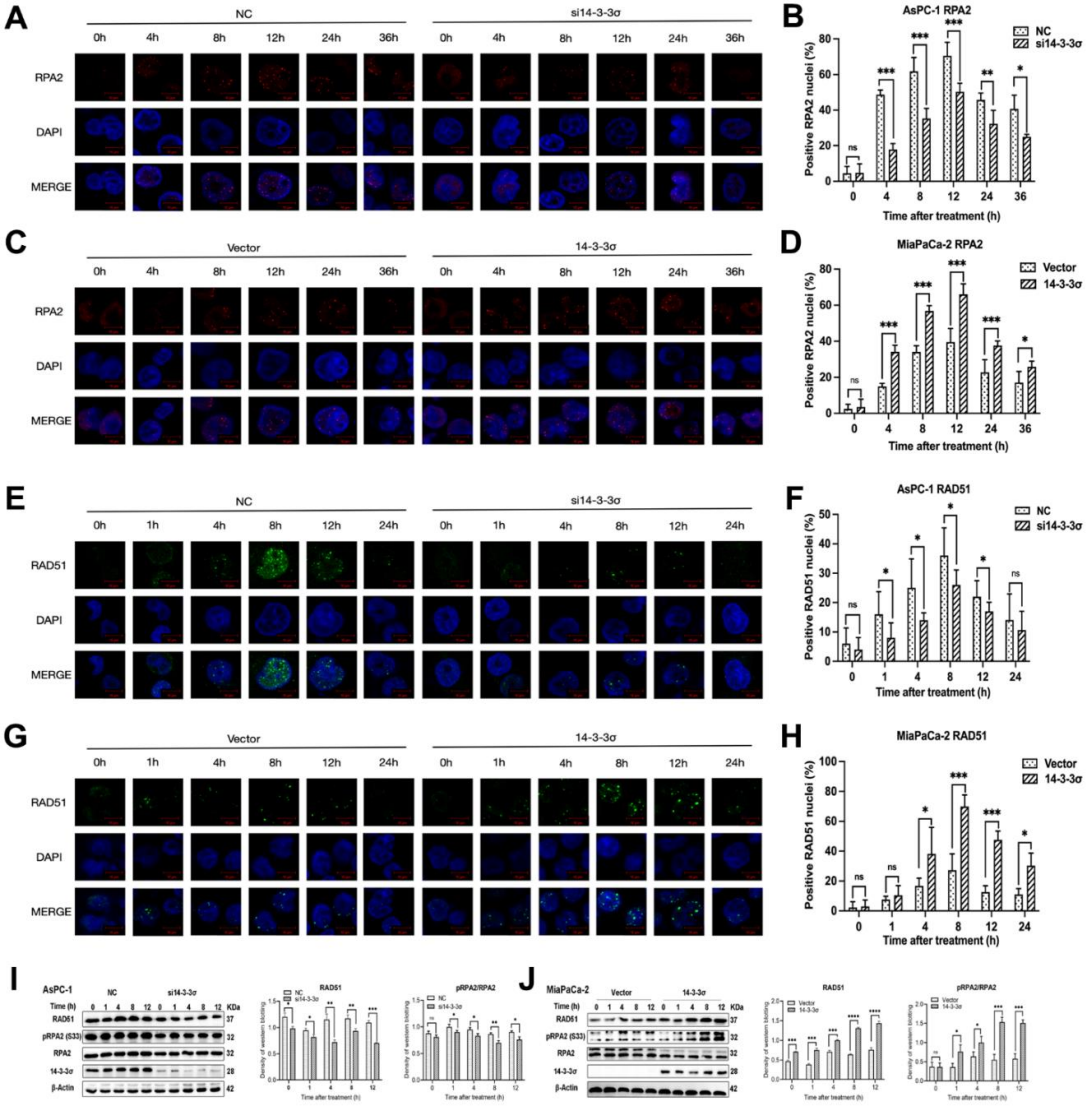
**14-3-3σ enhances the HR pathway activation.** (**A**) The representative images of the RPA2 foci in AsPC-1 cells after 3 Gy carbon ion irradiation in different groups were analyzed by immunofluorescence (IF) staining. (**B**) Percentage of positive nuclei of RPA2 in AsPC-1 cells in different groups (5 foci or more per nucleus being considered positive). (**C**) The representative images of the RPA2 foci in MiaPaCa-2 cells after 3 Gy carbon ion irradiation in different groups were analyzed by IF staining. (**D**) Percentage of positive nuclei of RPA2 in MiaPaCa-2 cells in different groups (5 foci or more per nucleus being considered positive). (**E**) The representative images of the RAD51 foci in AsPC-1 cells after 3 Gy carbon ion irradiation in different groups were analyzed by IF staining. (**F**) Percentage of positive nuclei of RAD51 in AsPC-1 cells in different groups (5 foci or more per nucleus being considered positive). (**G**) The representative images of the RAD51 foci in MiaPaCa-2 cells after 3 Gy carbon ion irradiation in different groups were analyzed by IF staining. (**H**) Percentage of positive nuclei of RAD51 in MiaPaCa-2 cells in different groups (5 foci or more per nucleus being considered positive). (**I**) Relative protein expression of RAD51, pRPA2, RPA2 in si14-3-3σ and NC AsPC-1 cells, 0 h, 1 h, 4 h, 8 h, and 12 h post-irradiation. (**J**) Relative protein expression of RAD51, pRPA2, RPA2 in 14-3-3σ-overexpression and vector MiaPaCa-2 cells, 0 h, 1 h, 4 h, 8 h, and 12 h post-irradiation. For IF, at least 100 cells per condition were analyzed. Data were presented as the mean ± standard deviation (SD). *p <0.05, **p <0.01, ***p <0.001, ****p <0.0001, ns: not significant. Scale bar = 10 μm.

Afterward, we performed immunofluorescence (IF) to detect the effect of 14-3-3σ on the recruitment kinetics of RAD51 and RPA2 at the DSB damage site, the two key repair factors of the HRR pathway for restoring clustered DSBs. The depletion of 14-3-3σ strongly inhibited the recruitment of RPA2—a single-strand-binding protein involved in the end excision of the HR process—to DNA damage sites, implying that 14-3-3σ contributes to the end excision step of HRR (Figure 7A, 7B). Notably, the recruitment of RAD51, the main recombinase related to HRR, was mitigated in si14-3-3σ AsPC-1 cells. In contrast, these foci in the NC group remained high in number or decreased dramatically (Figure 7E, 7F). The synergistic effect of overexpressed 14-3-3σ combined with carbon ions recruited more RAD51 and RPA2 foci than that by carbon ion irradiation alone, substantially enhancing the efficiency of the HR pathway (Figure 7C, 7D, 7G, 7H). RAD51, along with the phosphorylation of RPA, can reach DSB sites early in the HR pathway. Hyperphosphorylated RPA guarantees that RAD51 interacts with RPA with higher affinities than the unphosphorylated RPA [37]. Similarly, we found that RAD51 and phosphorylation of RPA2 after carbon ion irradiation were attenuated in the 14-3-3σ depletion AsPC-1 cells and increased in the 14-3-3σ proficient group, as evident from immunoblotting results (Figure 7I, 7J). We concluded that 14-3-3σ improves the accumulation of RPA2 and RAD51, thus guaranteeing the completion of carbon ion irradiation induced HRR.

### HRR pathway inhibitor abolishes 14-3-3σ-mediated carbon ion irradiation resistance

**Figure 8:**
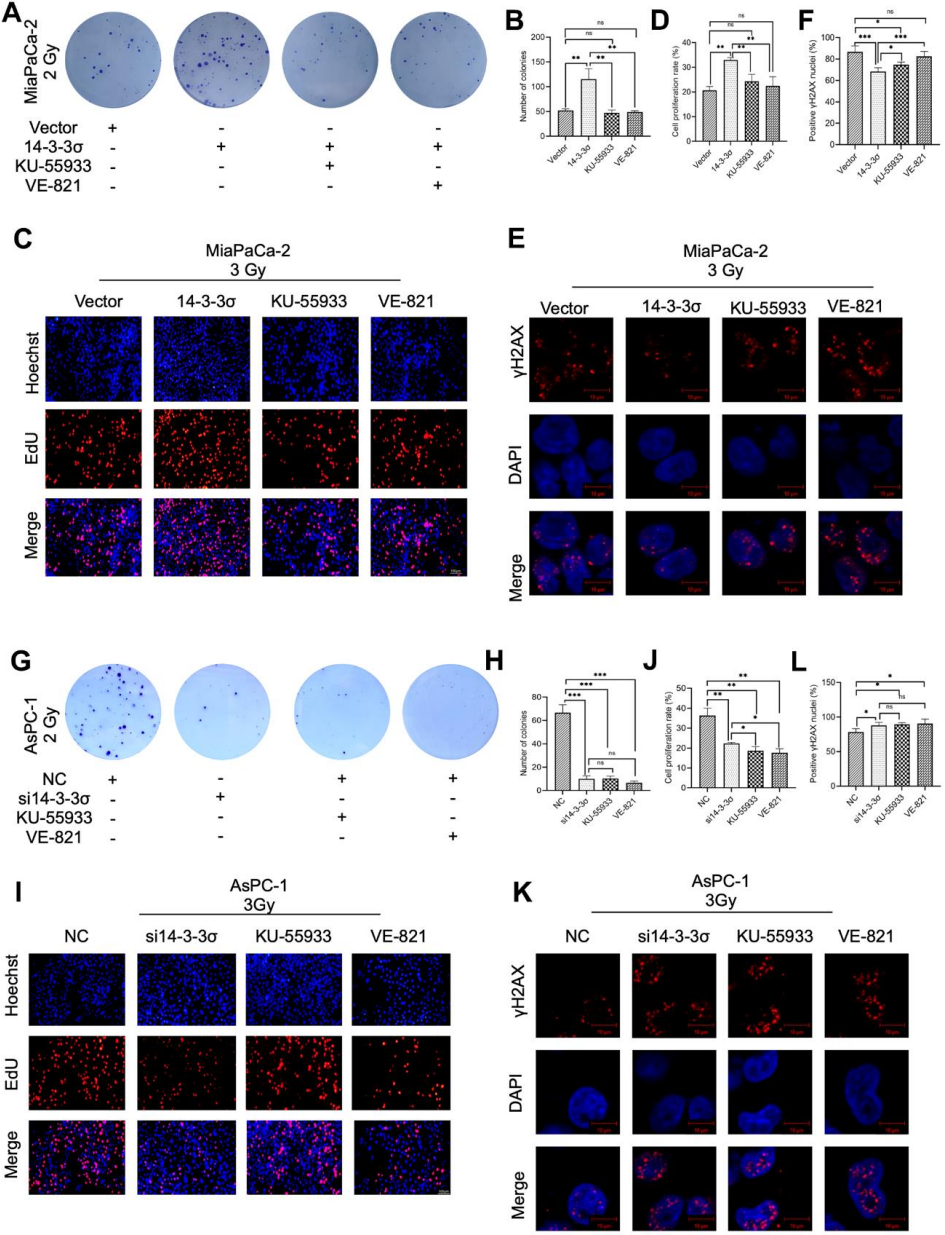
**Pharmacological inhibition of the HR pathway abolishes 14-3-3σ-mediated carbon ion irradiation resistance.** Colony formation assay (**A**, **B**), EdU assay (**C**, **D**), and immunofluorescence (IF) staining of γH2AX 1 h (**E**, **F**) in MiaPaCa-2 cells after 2 Gy carbon ion irradiation in different groups: vector group (empty vector), 14-3-3σ group (14-3-3σ overexpression), KU-55933 group (14-3-3σ overexpression + KU-55933 [inhibitor of ATM, 10μM, dissolved in DMSO]), and VE-821 group (14-3-3σ overexpression + VE-821 [inhibitor of ATR, 10μM, dissolved in DMSO]). Colony formation assay (**G**, **H**), EdU assay (**I**, **J**), and IF staining of γH2AX 1 h (**K**, **L**) in AsPC-1 cells after 2 Gy carbon ion irradiation in different groups: NC group (negative control), si14-3-3σ group (14-3-3σ knockdown), KU-55933 group (NC + KU-55933 [inhibitor of ATM, 10μM, dissolved in DMSO]), and VE-821 group (NC + VE-821 [inhibitor of ATR, 10μM, dissolved in DMSO]). For IF, at least 100 cells per condition were analyzed. Data were presented as the mean ± standard deviation (SD) (n=3); *p <0.05, **p <0.01, ***p <0.001, ****p <0.0001, ns: not significant. For EdU, scale bar = 100 μm; For IF, scale bar = 10 μm.

The correlation between 14-3-3σ and the HR pathway was elucidated by investigating whether 14-3-3σ-induced carbon ion radioresistance could abrogate the blocking of the HR signaling pathway. The ATR inhibitor VE-821 and ATM inhibitor KU-55933 were added to the 14-3-3σ overexpression group following carbon ion irradiation (VE-821 group, 14-3-3σ overexpression + VE-821, KU-55933 group, 14-3-3σ overexpression + KU-55933, respectively). The other two groups in this experiment were the Vector group (empty vector + DMSO) and the 14-3-3σ group (14-3-3σ overexpression + dimethyl sulfoxide [DMSO]). We first seeded 3000 cells/well to observe the colony formation ability in these four groups. As shown in Figure 8A, 8B, 14-3-3σ overexpression promoted the 2 Gy carbon ion irradiation colony formation of PAAD cells, and as expected, ATR or ATM blocking almost eliminated this phenomenon. In addition, these two inhibitors exerted equal effects. We next performed the EdU assay; compared with the Vector group, 14-3-3σ overexpression significantly enhanced the proliferation ability (proliferation rate: Vector group versus 14-3-3σ group: 20.67%±1.57% versus 32.99%±0.94%). The proliferation ability decreased with the addition of ATR inhibitor or ATM to MiaPaCa-2 cells (proliferation rate: VE-821 group: 22.48%±3.71%, KU-55933 group: 24.30%±2.86%), which exerted no statistical difference in comparison with the Vector group (Figure 8C, 8D). Finally, we studied the accumulation of γH2AX 1 h after 3 Gy carbon ion radiation. 14-3-3σ overexpression significantly decreased the formation of γH2AX signals; the γH2AX foci increased with the activation of ATR or ATM. Furthermore, the ATR pathway blocking exerted a more significant effect on 14-3-3σ-induced γH2AX accumulation than the ATM pathway blocking (Figure 8E, 8F). These data demonstrated that blocking the HR pathway, either ATR or ATM pathway, eliminated 14-3-3σ overexpression-mediated carbon ion irradiation resistance. To thoroughly verify the hypothesis that 14-3-3σ induced carbon ion radioresistance through the HR pathway, we detected the influence of 14-3-3σ depletion and ATR or ATM activation inhibition on carbon ion irradiation resistance. We created the following four groups for the subsequent studies: NC group (NC + DMSO), si14-3-3σ group (si14-3-3σ + DMSO), VE-821 group (NC + VE-821), and KU-55933 group (NC + KU-55933). The colony forming (Figure 8G, 8H) and EdU assays (Figure 8I, 8J) revealed that either the depletion of 14-3-3σ or the blocking of either ATR or ATM reduced the proliferation ability along with the radiation of carbon ion radiation. The accumulation γH2AX 1 h after 3 Gy carbon ions significantly increased in the si14-3-3σ group, VE-821 group, and KU-55933 group in comparison with the NC group (Figure 8K, 8L). Above data indicated that the 14-3-3σ could be the upstream member of the HR pathway, and both 14-3-3σ depletion and ATR/ATM inhibition could be used as therapeutic targets for PAAD carbon ion radiotherapy.

### 14-3-3σ promotes carbon ion irradiation resistance of pancreatic cancer *in vivo*

**Figure 9:**
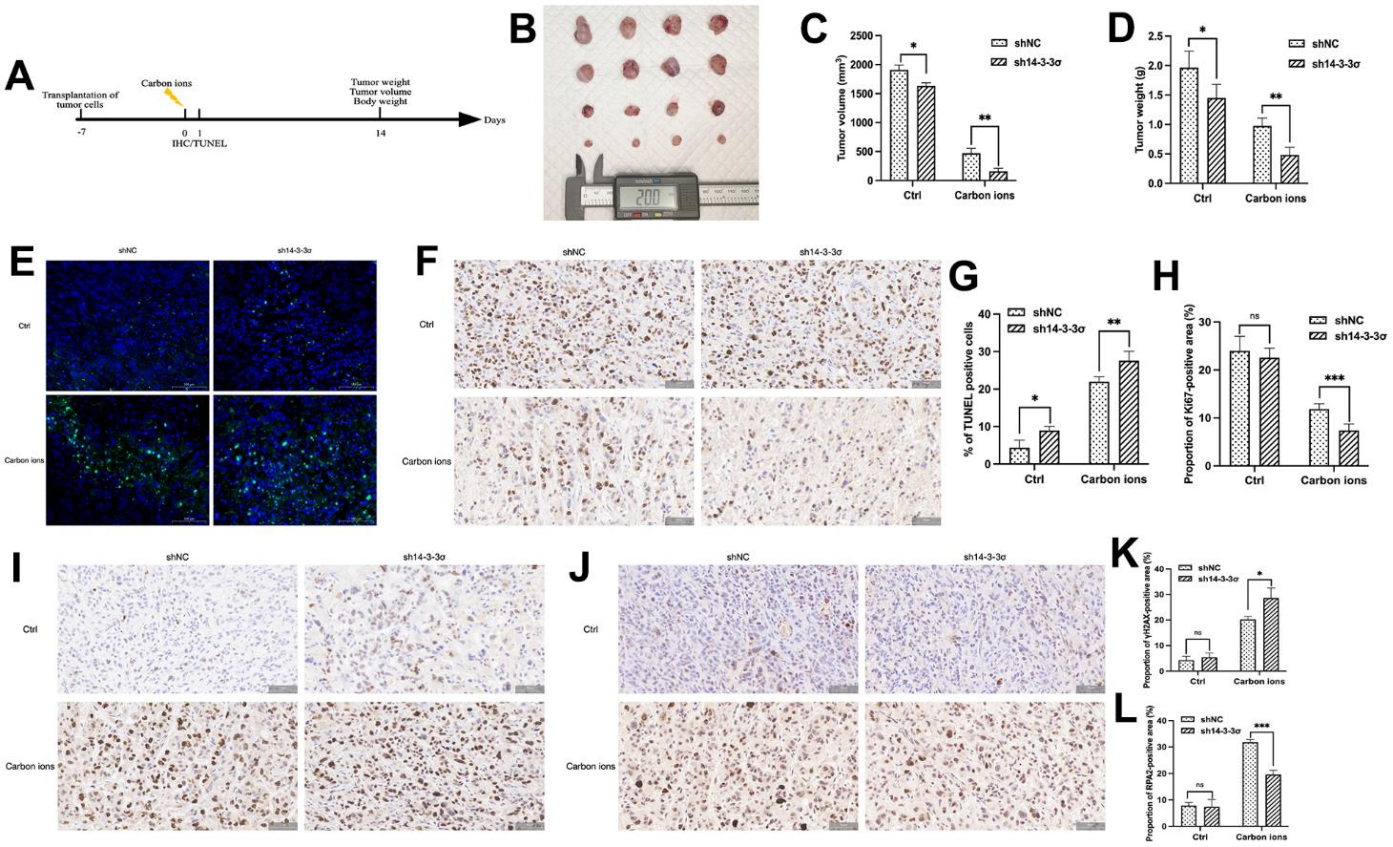
**14-3-3σ promotes the carbon ion radiosensitivity of PAAD cells *in vivo*.** (**A**) The overall design of the *in vivo* experiments. (**B**–**D**) Macro images, tumor volume, and tumor weight of the excised tumors for each group (4 mice/group). (**E**, **F**) Representative 40× images of TUNEL immunofluorescence (IF) staining and immunohistochemical (IHC) staining of Ki67 of tumor tissue in sections in different groups at 24 h after 5 Gy carbon ion irradiation. (**G**, **H**) Respective percentage of TUNEL-positive cells in the excised tumor by quantitative analysis. (**H**) Respective percentage of Ki67-positive area in the excised tumor by quantitative analysis. (**I**, **J**) Representative 40× images of IHC staining of γH2AX, and RPA2 of tumor tissue in sections in different groups at 24 h after 5 Gy carbon ion irradiation. (**K**, **L**) Respective percentage of γH2AX-, RPA2-positive area in the excised tumor by quantitative analysis. Data were presented as the mean ± standard deviation (SD); *p <0.05, **p <0.01, ***p <0.001, ****p <0.0001, ns: not significant. Scale bar = 50 μm.

The above results demonstrated that 14-3-3σ promoted carbon ion radioresistance by hindering the HRR pathway. We constructed the stably 14-3-3σ-deficient cells using lentivirus containing 14-3-3σ short hairpin RNA (shRNA) (Supplementary Figure 2A–2C) to study the effect of 14-3-3σ on carbon ion irradiation *in vivo*. We generated subcutaneous PAAD xenograft models using AsPC-1 cells (Figure 9A). As depicted in Figure 9B–9D, the sh14-3-3σ group inhibited xenografts in terms of size, weight, and volume, and the inhibition was more significant than that following carbon ion irradiation. This was in line with the *in vitro* outcomes. The Ki67 IHC and terminal deoxynucleotidyl transferase dUTP nick end labeling (TUNEL) IF staining were performed to detect the proliferation and apoptosis rate of tumor cells. Tumors derived from sh14-3-3σ group displayed fewer proliferating cells (NC group: 24.00%±3.02%, sh14-3-3σ group: 22.58%±1.99%, carbon ion group: 11.83%±1.15%, combination group: 7.40%±1.33%) (Figure 9E, 9G), and more cell apoptosis (NC group: 4.40%±1.99%, sh14-3-3σ group: 8.95%±1.05%, carbon ion group: 21.97%±1.30%, combination group: 27.62%±2.47%) (Figure 9F, 9H). In addition, the combination group owned more γH2AX-positive cells, a marker of unrepaired DNA damage, than the carbon ion irradiation one (Figure 9I, 9K). Finally, we detected RPA2-positive cells and found that sh14-3-3σ reduced the carbon-induced number of RPA2-positive cells (Figure 9J, 9L). The above outcomes indicated that 14-3-3σ depletion suppressed RPA2 expression, thus inhibiting cell proliferation to facilitate carbon ion radiosensitivity of PAAD cells *in vivo*.

## DISCUSSION

PAAD is a highly common and lethal cancer, whose increasing incidence and mortality have resulted in a substantial economic burden worldwide [2, 38]. At present, radiotherapy is the mainstay treatment of tumors. However, the extreme hypoxic environment of PAAD cells limits the clinical benefits of radiotherapy. In this regard, carbon ion radiotherapy (CIRT) can cause more direct DNA damage and create DSBs theoretically due to its high relative biological effectiveness (RBE). Its feature of oxygen independence improves its effect against hypoxia-resistant and radioresistant tumors such as PAAD [39]. Our study confirmed that 14-3-3σ was highly expressed in PAAD tissues and predicted its poor survival. 14-3-3σ enhanced the radioresistance of carbon ion-irradiated PAAD cells by boosting the recruitment of RAD51 and RPA2 and subsequently hindering the HRR pathway.

Jung et al. [23] reported that multiple tumors express abnormal 14-3-3σ; for example, 14-3-3σ mediates tumor progression and metastasis in gastric cancer. The knockdown of 14-3-3σ inhibited the growth and metastasis of hepatocellular carcinoma in BALB/c nude mice [24]. Our experiments indicated that the knockdown of 14-3-3σ impeded the proliferation and enhanced the percentage of apoptosis of PAAD cells by *in vitro* and *in vivo* experiments, which were in accordance with previous studies [25, 26].

The arrest of the cell cycle is indispensable to DDR and maintaining genome stability. 14-3-3σ is critical for continuous G2/M arrest following DNA damage. The GSEA analysis indicated that the high expression of 14-3-3σ was associated with the activation of the cell cycle in PAAD (Figure 6A). Flow cytometry revealed that 14-3-3σ arrested PAAD cells in the G2/M phase, during which homologous sister chromatids are formed, and the HR-associated proteins are increased [22, 40], ensuring that the cells accurately repair DNA damage and survive in DNA damage treatment, thereby contributing to carbon ion irradiation resistance. Similarly, 14-3-3σ can sequestrate the CDK1/cyclin B complex, thus maintaining cells in the G2 phase, which can induce G2/M checkpoint activation [41]. Evidence indicated that 14-3-3σ interacts with RNF126 to promote the G2 arrest in a p53-independent manner [42]. On the other hand, there is also a study that 14-3-3σ promotes radioresistance by blocking PAAD cells in the G2/M phase after IR and thus enhances the NHEJ repair efficiency of X-ray-induced DSBs [25].

Carbon ions have been reported to induce rather complex or clustered DSBs than those by X-rays, which are generally unrepairable [43–46]. 53BP1 colocalizes with γH2AX at all damaged sites and is essential in NHEJ in most cell lines. It is reported that 53BP1 is located at the DNA damage site to initiate HR through RAD51 [47]. We found a radiosensitizing effect following 14-3-3σ depletion, verified by restrained activity of the HR pathway and abundance of unpaired DNA damage, demonstrated by measuring γH2AX and 53BP1 levels. RAD51 is a vital member of the HR pathway, facilitating high-precision DNA damage repair by locating the homology in sister chromatids and enabling DNA strand invasion into sister chromatids [48]. Furthermore, the decreased expression of RPA2 in 14-3-3σ depletion cells after carbon ions were confirmed *in vivo*, which further confirmed our conclusion.

DSB resection is a vital step in the overall DDR [15]. Once DSB induces, MRN/Sae2-CTIP and Exo1/Dna2-dependent DSB end resection form the ssDNA regions, 14-3-3 protein can directly bind to the central domain of Exo1 and limit its association with DDR and the DNA resection process [15, 49, 50]. Human Exo1 coactions with at least six of the seven 14-3-3 human paralogs, including 14-3-3σ [49]. The deletion of 14-3-3-mediated regulation of Exo1 can induce the excessive DNA resection at the damage site, which attenuates the HR and DSB repair, leading to chromosomal instability [51]. Thus, we supposed that 14-3-3σ depletion expanded the process of the DNA-end resection by enhancing the activation of Exo1, therefore altered the recruitment of both RPA2 and RAD51, and eventually decreasing the repair of HR-mediated clustered DSBs in 14-3-3σ-depletion cells irradiated with carbon ions.

Multiple signal transduction processes orchestrate DDRs, prominent of which are ATR–Chk1 and ATM–Chk2 pathways. Our study demonstrated that the use of ATR/ATM inhibitor could remove the carbon ion resistance induced by 14-3-3σ overexpression. These two pathways are usually activated simultaneously following the exposure of cells to different genotoxic damage, such as irradiation and cytotoxic chemotherapy [12]. The ATR–Chk1 pathway can sense ssDNA produced during DSB processing [52, 53]. ATM can phosphorylate and activate Chk2, which subsequently separates from damaged sites and disperses as a monomer throughout the nucleus to function on multiple processes, including cell cycle progression, apoptosis, and gene transcription [54]. Numerous studies have demonstrated that RAD51 replacing RPA in resected ssDNA is critically dependent on Chk1-mediated phosphorylation of BRCA2 and RAD51 [18, 55]. Reduced levels of phosphorylated Chk1 are a typical marker of impaired HR efficiency [17]. Our study found that the ATM and ATR mRNA were reduced after the 14-3-3σ depletion. Meanwhile, the phosphorylation of both Chk1 and Chk2 decreased after carbon ion irradiation in the 14-3-3σ-deficient AsPC-1 cells. Similar results were reported that the phosphorylation of Chk1 after irradiation is related to Chk1 binding to 14-3-3σ [56]. Together, we speculated that 14-3-3σ perhaps functions on multiple sites in the process of DSB resections for generating ssDNA for HR.

As far as we know, our study firstly delineated the role of 14-3-3σ in carbon ion-induced DNA damage and radiosensitivity of PAAD by regulating HR-mediated DNA damage repair. However, our research is confined to *in vitro* and *in vivo* experiments, and clinical samples are warranted. It is essential to elucidate the repair pathways and accurate carbon ion irradiation repair products. As 14-3-3σ is an adaptor molecule [57], which may interact with other molecules to regulate the process of HRR pathway, nevertheless, the current experimental results cannot distinguish the direct target of 14-3-3σ following DNA damage repair and signaling, and further experimental evidence is required.

## CONCLUSIONS

**Figure 10:**
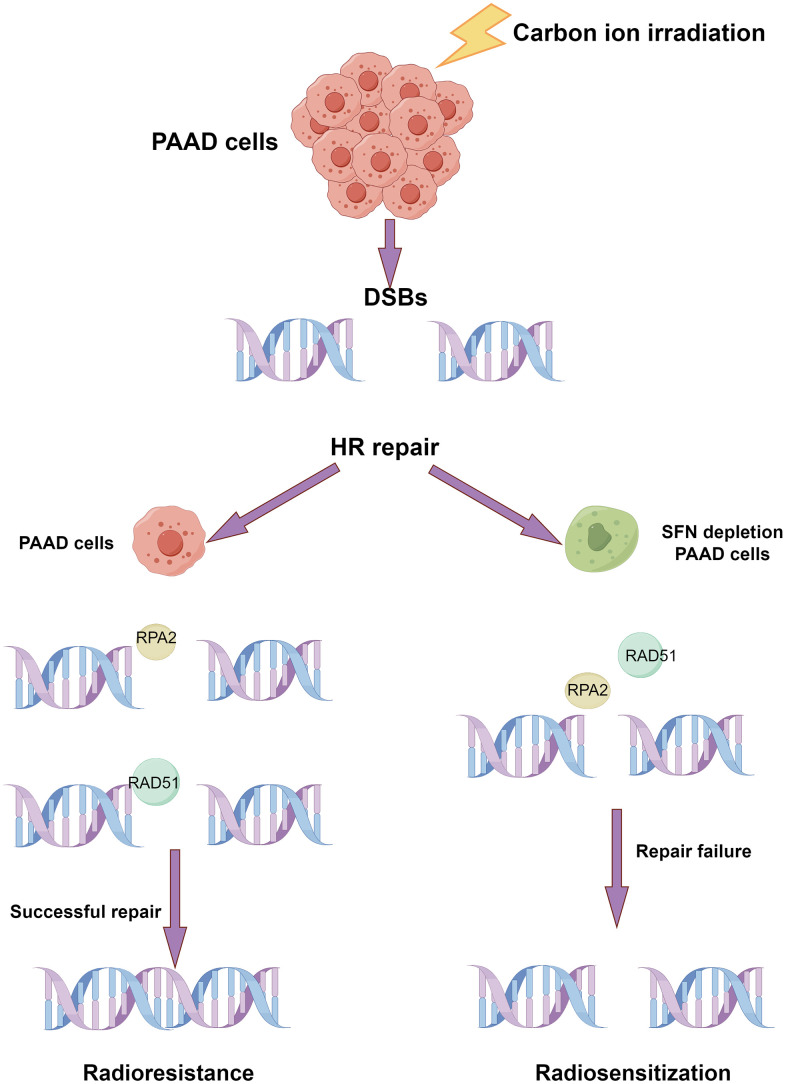
**Schematic diagram of the possible mechanism that the depletion of 14-3-3σ enhances the sensitivity of carbon ion irradiation.** (By Figdraw).

14-3-3σ depletion affects the cellular behaviors of PAAD cells following carbon ion irradiation, including proliferation inhibition, cell cycle arrest reduction, and DNA damage induction. The xenograft model implicated 14-3-3σ in a carbon ion resistance role in PAAD cells. 14-3-3σ reduced the effect of carbon ion irradiation by promoting HRR of DNA damage by negatively regulating DNA end resection (Figure 10). Therefore, 14-3-3σ is a candidate target to strengthen the radiosensitivity of PAAD to carbon ions.

## MATERIALS AND METHODS

### Bioinformatic analysis

The “stats” R package was used to conduct the PCA. We used the SangerBox3.0 [58] to download the pan-cancer gene expression data via TCGA dataset and evaluated the pan-cancer expression of 14-3-3σ. The Kaplan-Meier curves of OS, DSS, PFS, and DFS were plotted using the Sangbox3.0 tool “prognostic analysis” module to assess the underlying prognostic value of the *14-3-3σ* gene in PAAD. We used the “clinical stages and gene expression analysis” module to identify the relationship between the 14-3-3σ mRNA expression and the grade/stage of PAAD. We obtained the PAAD data from the TCGA GDC website [59] and assessed the relevance between 14-3-3σ and clinical features using the univariate logistic regression analysis. The GSEA was carried out to investigate the 14-3-3σ-relative biological signaling pathway in PAAD. Finally, we examined the protein expression of 14-3-3σ in PAAD and normal tissues using the HPA (http://www.proteinatlas.org/) database.

### Cell culture and irradiation treatment

AsPC-1, hTERT-HPNE, MiaPaCa-2, PANC-1, and SW1990 cells were acquired from the American Type Culture Collection (ATCC) (Manassas, NY, USA), and maintained in Dulbecco’s modified Eagle’s medium (DMEM) supplemented with 10% fetal bovine serum (FBS) in humidified incubators of 5% CO_2_ at 37° C. The Heavy Ion Medical Machine (HIMM) at Lanzhou Heavy Ion Hospital provided the heavy ion beam with an energy value of 120 MeV/u and linear energy transfer of 80 keV/μm at the center of the spread-out Bragg peak. The dose rate was 2 Gy/min.

### Knockdown and overexpression of 14-3-3σ in PAAD cells

Specifically, targeted small interfering RNA (siRNA) and overexpression plasmids were designed and synthesized by GenePharma (Suzhou, China) to knock down and overexpress 14-3-3σ, respectively. The siRNAs and plasmids were transfected into AsPC-1 and MiaPaCa-2 cells using GP-transfect-Mate (GenePharma; Suzhou, China) and Lipo8000™ (Beyotime; Shanghai, China), respectively. The experiments were conducted at 48 h post-transfection. Lentiviruses containing 14-3-3σ shRNA were acquired from Suzhou GenePharma Co., Ltd. The targeting siRNAs, shRNA, and plasmid sequences are listed in Supplementary Table 3.

### Protein isolation and immunoblotting

The total proteins were obtained at anticipated time points from PAAD cells using radio immunoprecipitation assay (RIPA) buffer (Solarbio, Beijing, China) supplemented with phenylmethylsulfonyl fluoride (PMSF) and phosphatase inhibitors. We used the bicinchoninic acid (BCA) kit (Solarbio, Beijing, China) to quantify the proteins. The proteins were separated by sodium dodecyl sulfate–polyacrylamide gel electrophoresis (SDS-PAGE) and transferred to polyvinylidene fluoride membranes (Immobilon-P, Ireland). After blocking in 5% skim milk for 2 h (room temperature), the membranes were immunoblotted with primary antibodies (Supplementary Table 4) at 4° C overnight and subsequently incubated with secondary antibodies at room temperature for 1 h and examined using a chemiluminescence imaging system (QuickChemi5200; Monad, China). The ImageJ software was adopted to quantify the relative protein expression.

### RNA isolation and qRT-PCR

The total RNA was obtained from PAAD cells by the usage of the TRIzol (Invitrogen, Waltham, MA, USA) reagent. The RNA samples were used for complementary DNA synthesis using the Hifair^®^ III 1st Strand cDNA Synthesis Supermix reverse transcription kit for qPCR (gDNA digester plus) (Yeasen, Shanghai, China). Next, the qRT-PCR was completed using the Hieff^®^ qPCR SYBR^®^ Green Master Mix kit (Yeasen, Shanghai, China) according to the manufacturer’s instructions. The results were analyzed using the 2^-∆∆Ct^ method with β-actin as the housekeeping gene. The primer sequences are listed in Supplementary Table 5.

### Colony forming assay and cell proliferation assay

Single cells (400–5000 cells per well) were inoculated into 6-well plates and cultivated overnight, after carbon ion irradiation (0–3 Gy), the cells were placed in the incubator for 10 to 14 days to form colonies. We fixed and stained the plates using methanol for 20 min and crystal violet for 15 min. Afterward, we counted the number of colonies (at least 50 cells) under a light microscope (Olympus, Japan). The survival curve was plotted using the linear–quadratic equation: surviving fraction $(SF)=e^{\left(-(\alpha D+\beta D^{2}\right)}$.

We used the EdU assay kit (RiBoBio, Guangdong, China) to measure the proliferation rate of cells and acquired images under a fluorescence microscope (Olympus, Tokyo, Japan). We utilized the CCK8 assay to check the proliferation of PAAD cells. Cells (2 ×10^3^ cells/well) were inoculated into 96-well plates after carbon ion irradiation. After 24, 48, and 72 h of incubation, 20 μL of the CCK8 solution (Yeasen, Shanghai, China) was added to each well, followed by a 2 h incubation at 37° C. The absorbance at 450 nm was obtained using a microplate reader (Tecan Infinite 200 M; Männedorf, Switzerland).

### Apoptosis assays

Apoptotic cell death was assessed using a TUNEL apoptosis assay kit (Beyotime, Shanghai, China). Firstly, the cells were inoculated in the 12-well after 48 h transfection, after 24 h of 3Gy carbon ion irradiation, we immobilized the cells using 4% paraformaldehyde. Then the phosphate-buffered saline (PBS) containing 0.3% Triton X-100 was used to incubate cells for 5 min at room temperature. Finally, 50 μL of TUNEL staining solution was added to every well and incubated at 37° C in the dark for 1 h. Cells with red fluorescence were defined as apoptotic cells using a fluorescence microscope (Leica, Wetzlar, Germany). We used the Image J software to count the positive cells.

### Flow cytometry for cell cycle

The cell cycle was studied by first fixing the harvested cells with 75% ethanol overnight at −20° C. At the time of testing (6 h, 12 h, and 24 h after irradiation), the fixed cells were rehydrated with PBS for 15 min and centrifuged, and subsequently treated with 500 μL of the DNA staining solution (away from light, at room temperature for 30 min) (Yeasen, Shanghai, China) and tested on the IDEAS Application v6.0 software at −20° C overnight. The data were calculated using the FlowJo-V10.8.1 software. We collected 15,000 cells for the cell cycle for each sample.

### Immunofluorescence (IF) microscopy

The cells were inoculated onto Φ20 mm glass bottom cell culture dishes and cultivated overnight. Next, the cells were irradiated with carbon ions (3 Gy) and immobilized using 4% paraformaldehyde for 20 min at the indicated times (0, 1, 4, 8, 12, and 24 h after irradiation). Afterward, the cells were permeabilized with PBS containing 0.5% Triton X-100 (Solarbio; Beijing, China) on ice for 15 min, and blocked with 10% goat normal serum in PBS for another 1 h. Afterward, the cells were probed with antibodies against γH2AX, 53BP1, RAD51, and RPA2 (Supplementary Table 4) at 4° C overnight and incubated with secondary goat anti-mouse IgG (H&L) antibody–Alexa Fluor 594 or goat anti-rabbit IgG (H&L) antibody–Alexa Fluor 488 (Immunoway; Jiangsu, China) at 1:200 dilution at room temperature for 1 h in the dark. The nuclei were stained with 4’, 6-diamidino-2-phenylindole (DAPI; Solarbio, Beijing, China). The images were acquired using a confocal microscope (Carl Zeiss; Jena, Germany). We analyzed no less than 100 cells per condition to quantify IF experiments.

### Drug treatment

VE-821 (HY-14731), an ATR inhibitor, and KU-55933 (HY-12016), an ATM inhibitor, were purchased from MedChemExpress (Monmouth Junction, NJ, USA). Exponentially growing cells were preincubated with VE-821 (10 μM, dissolved in dimethyl sulfoxide [DMSO]) or KU-55933 (10 μM, dissolved in DMSO) for 2 h before conducting the experiments.

### Animal studies

Male BALB/c nude mice (4–5 weeks old) were purchased from SPF (Beijing) Biotechnology Co., Ltd., and maintained in a specific pathogen-free environment (24±2° C, relative humidity of 55±5%, and a 12 h light/dark cycle) with free access to standard laboratory chow and water at the Gansu University of Chinese Medicine. We divided the mice into four groups (8 mice/group): control group, 14-3-3σ-shRNA group, carbon ion group, and carbon ion + 14-3-3σ-shRNA group (combination group). AsPC-1 cells or 14-3-3σ-shRNA AsPC-1 cells (5 × 10^6^ cells in 100 μL of PBS/mouse) were subcutaneously inoculated into the right flank regions of mice. Once tumors reached a volume of ~100 mm^3^, mice in the carbon ion group and combination group were vertically irradiated with 5 Gy carbon ions, the beams were provided by the HIMM. Four mice in each group were randomly selected and sacrificed 24 h after irradiation, and we measured the tumor volume of the remaining mice weekly until 28 days after irradiation, and subsequently sacrificed. Tumor weight, tumor volume, and body weight were determined. The formula of (length × width^2^)/2 was used to calculate the tumor volumes.

### IHC and TUNEL staining

The tumors were fixed in 4% paraformaldehyde and then embedded in paraffin, 3 μm sections were sliced after the tissues were dewaxed, rehydrated, and incubated for antigen retrieval. For the IHC assay, after blocking with goat serum, the sections were incubated with primary antibody (Supplementary Table 4), followed by PBS rinses. After incubation with the corresponding secondary antibody, the sections were incubated with diaminobenzidine (DAB). The proportion of positive area was analyzed using ImageJ software. For TUNEL staining, the one-step TUNEL kit (Elabscience; Wuhan, China) was used as per the manufacturer’s instructions. The number of green fluorescent cells indicated positive cells. Four sections were selected in each group, and four fields were randomly counted per section using a fluorescence microscope (3DHISTECH, Budapest, Hungary).

### Statistical analyses

We used the SPSS software (Chicago, IL, USA) for statistical analysis and GraphPad Prism 9 (GraphPad Software, Inc., San Diego, CA, USA) for constructing the graphs. Quantitative results are represented as means ± standard deviations (SD). Student’s *t*-test was used for a single comparison; a one-way analysis of variance followed by the Newman-Keuls multiple comparison test was used to compare more than two groups. All results originated from at least three independent experiments. *P* < 0.05 was considered significant.

## Supplementary Material

Supplementary Figures

Supplementary Tables

## References

[1] Owens DK, Davidson KW, Krist AH, Barry MJ, Cabana M, Caughey AB, Curry SJ, Doubeni CA, Epling JW Jr, Kubik M, Landefeld CS, Mangione CM, Pbert L, et al, and US Preventive Services Task Force. Screening for Pancreatic Cancer: US Preventive Services Task Force Reaffirmation Recommendation Statement. JAMA. 2019; 322:438–44. 10.1001/jama.2019.10232 31386141

[2] Hu JX, Zhao CF, Chen WB, Liu QC, Li QW, Lin YY, Gao F. Pancreatic cancer: A review of epidemiology, trend, and risk factors. World J Gastroenterol. 2021; 27:4298–321. 10.3748/wjg.v27.i27.4298 34366606 PMC8316912

[3] Rawla P, Sunkara T, Gaduputi V. Epidemiology of Pancreatic Cancer: Global Trends, Etiology and Risk Factors. World J Oncol. 2019; 10:10–27. 10.14740/wjon1166 30834048 PMC6396775

[4] Park W, Chawla A, O’Reilly EM. Pancreatic Cancer: A Review. JAMA. 2021; 326:851–62. 10.1001/jama.2021.13027 34547082 PMC9363152

[5] Liermann J, Shinoto M, Syed M, Debus J, Herfarth K, Naumann P. Carbon ion radiotherapy in pancreatic cancer: A review of clinical data. Radiother Oncol. 2020; 147:145–50. 10.1016/j.radonc.2020.05.012 32416281

[6] Baltazar F, Tessonnier T, Haberer T, Debus J, Herfarth K, Tawk B, Knoll M, Abdollahi A, Liermann J, Mairani A. Carbon-ion radiotherapy (CIRT) as treatment of pancreatic cancer at HIT: initial radiation plan analysis of the prospective phase II PACK-study. Radiother Oncol. 2023; 188:109872. 10.1016/j.radonc.2023.109872 37634764

[7] Hagiwara Y, Yamada S, Isozaki Y, Takiyama H, Shinoto M, Kawashiro S, Bhattacharyya T, Nemoto K, Tsuji H. Efficacy and feasibility of re-irradiation using carbon ions for pancreatic cancer that recurs after carbon-ion radiotherapy. Clin Transl Radiat Oncol. 2020; 26:24–9. 10.1016/j.ctro.2020.10.007 33294643 PMC7691119

[8] Zhang Q, Karnak D, Tan M, Lawrence TS, Morgan MA, Sun Y. FBXW7 Facilitates Nonhomologous End-Joining via K63-Linked Polyubiquitylation of XRCC4. Mol Cell. 2016; 61:419–33. 10.1016/j.molcel.2015.12.010 26774286 PMC4744117

[9] Park Y, Peoples AR, Madugundu GS, Sanche L, Wagner JR. Side-by-side comparison of DNA damage induced by low-energy electrons and high-energy photons with solid TpTpT trinucleotide. J Phys Chem B. 2013; 117:10122–31. 10.1021/jp405397m 23909580 PMC3817083

[10] Shibata A, Jeggo PA. DNA double-strand break repair in a cellular context. Clin Oncol (R Coll Radiol). 2014; 26:243–9. 10.1016/j.clon.2014.02.004 24630811

[11] Nikitaki Z, Velalopoulou A, Zanni V, Tremi I, Havaki S, Kokkoris M, Gorgoulis VG, Koumenis C, Georgakilas AG. Key biological mechanisms involved in high-LET radiation therapies with a focus on DNA damage and repair. Expert Rev Mol Med. 2022; 24:e15. 10.1017/erm.2022.6 35357290

[12] Smith J, Tho LM, Xu N, Gillespie DA. The ATM-Chk2 and ATR-Chk1 pathways in DNA damage signaling and cancer. Adv Cancer Res. 2010; 108:73–112. 10.1016/B978-0-12-380888-2.00003-0 21034966

[13] Mao Z, Bozzella M, Seluanov A, Gorbunova V. Comparison of nonhomologous end joining and homologous recombination in human cells. DNA Repair (Amst). 2008; 7:1765–71. 10.1016/j.dnarep.2008.06.018 18675941 PMC2695993

[14] Mazón G, Mimitou EP, Symington LS. SnapShot: Homologous recombination in DNA double-strand break repair. Cell. 2010; 142:646. 10.1016/j.cell.2010.08.006 20723763

[15] Chen X, Kim IK, Honaker Y, Paudyal SC, Koh WK, Sparks M, Li S, Piwnica-Worms H, Ellenberger T, You Z. 14-3-3 proteins restrain the Exo1 nuclease to prevent overresection. J Biol Chem. 2015; 290:12300–12. 10.1074/jbc.M115.644005 25833945 PMC4424361

[16] Mimitou EP, Symington LS. DNA end resection: many nucleases make light work. DNA Repair (Amst). 2009; 8:983–95. 10.1016/j.dnarep.2009.04.017 19473888 PMC2760233

[17] Chandler BC, Moubadder L, Ritter CL, Liu M, Cameron M, Wilder-Romans K, Zhang A, Pesch AM, Michmerhuizen AR, Hirsh N, Androsiglio M, Ward T, Olsen E, et al. TTK inhibition radiosensitizes basal-like breast cancer through impaired homologous recombination. J Clin Invest. 2020; 130:958–73. 10.1172/JCI130435 31961339 PMC6994133

[18] Gupta P, Saha B, Chattopadhyay S, Patro BS. Pharmacological targeting of differential DNA repair, radio-sensitizes WRN-deficient cancer cells *in vitro* and *in vivo*. Biochem Pharmacol. 2021; 186:114450. 10.1016/j.bcp.2021.114450 33571504

[19] Zhang J, Si J, Gan L, Zhou R, Guo M, Zhang H. Harnessing the targeting potential of differential radiobiological effects of photon versus particle radiation for cancer treatment. J Cell Physiol. 2021; 236:1695–711. 10.1002/jcp.29960 32691425

[20] Zafar F, Seidler SB, Kronenberg A, Schild D, Wiese C. Homologous recombination contributes to the repair of DNA double-strand breaks induced by high-energy iron ions. Radiat Res. 2010; 173:27–39. 10.1667/RR1910.1 20041757

[21] Nakajima NI, Brunton H, Watanabe R, Shrikhande A, Hirayama R, Matsufuji N, Fujimori A, Murakami T, Okayasu R, Jeggo P, Shibata A. Visualisation of γH2AX foci caused by heavy ion particle traversal; distinction between core track versus non-track damage. PLoS One. 2013; 8:e70107. 10.1371/journal.pone.0070107 23967070 PMC3743843

[22] Shibata A, Conrad S, Birraux J, Geuting V, Barton O, Ismail A, Kakarougkas A, Meek K, Taucher-Scholz G, Löbrich M, Jeggo PA. Factors determining DNA double-strand break repair pathway choice in G2 phase. EMBO J. 2011; 30:1079–92. 10.1038/emboj.2011.27 21317870 PMC3061033

[23] Jung JY, Koh SA, Lee KH, Kim JR. 14-3-3 Sigma Protein Contributes to Hepatocyte Growth Factor-mediated Cell Proliferation and Invasion via Matrix Metalloproteinase-1 Regulation in Human Gastric Cancer. Anticancer Res. 2022; 42:519–30. 10.21873/anticanres.15510 34969762

[24] Song J, Liu Y, Liu F, Zhang L, Li G, Yuan C, Yu C, Lu X, Liu Q, Chen X, Liang H, Ding Z, Zhang B. The 14-3-3σ protein promotes HCC anoikis resistance by inhibiting EGFR degradation and thereby activating the EGFR-dependent ERK1/2 signaling pathway. Theranostics. 2021; 11:996–1015. 10.7150/thno.51646 33391517 PMC7738881

[25] Chen Y, Li Z, Dong Z, Beebe J, Yang K, Fu L, Zhang JT. 14-3-3σ Contributes to Radioresistance By Regulating DNA Repair and Cell Cycle via PARP1 and CHK2. Mol Cancer Res. 2017; 15:418–28. 10.1158/1541-7786.MCR-16-0366 28087741 PMC5380477

[26] Li Z, Dong Z, Myer D, Yip-Schneider M, Liu J, Cui P, Schmidt CM, Zhang JT. Role of 14-3-3σ in poor prognosis and in radiation and drug resistance of human pancreatic cancers. BMC Cancer. 2010; 10:598. 10.1186/1471-2407-10-598 21040574 PMC2991307

[27] Boudreau A, Tanner K, Wang D, Geyer FC, Reis-Filho JS, Bissell MJ. 14-3-3σ stabilizes a complex of soluble actin and intermediate filament to enable breast tumor invasion. Proc Natl Acad Sci USA. 2013; 110:E3937–44. 10.1073/pnas.1315022110 24067649 PMC3799319

[28] Rial SA, Shishani R, Cummings BP, Lim GE. Is 14-3-3 the Combination to Unlock New Pathways to Improve Metabolic Homeostasis and β-Cell Function? Diabetes. 2023; 72:1045–54. 10.2337/db23-0094 37471599 PMC10382651

[29] Zhang K, Huang Y, Shi Q. Genome-wide identification and characterization of 14-3-3 genes in fishes. Gene. 2021; 791:145721. 10.1016/j.gene.2021.145721 34010706

[30] Kolbeinsson HM, Chandana S, Wright GP, Chung M. Pancreatic Cancer: A Review of Current Treatment and Novel Therapies. J Invest Surg. 2023; 36:2129884. 10.1080/08941939.2022.2129884 36191926

[31] Melnik MK, Webb CP, Richardson PJ, Luttenton CR, Campbell AD, Monroe TJ, O’Rourke TJ, Yost KJ, Szczepanek CM, Bassett MR, Truszkowski KJ, Stein P, Van Brocklin MW, et al. Phase II trial to evaluate gemcitabine and etoposide for locally advanced or metastatic pancreatic cancer. Mol Cancer Ther. 2010; 9:2423–9. 10.1158/1535-7163.MCT-09-0854 20682649

[32] Sollazzo A, Brzozowska B, Cheng L, Lundholm L, Scherthan H, Wojcik A. Live Dynamics of 53BP1 Foci Following Simultaneous Induction of Clustered and Dispersed DNA Damage in U2OS Cells. Int J Mol Sci. 2018; 19:519. 10.3390/ijms19020519 29419809 PMC5855741

[33] Nakajima NI, Yamauchi M, Kakoti S, Cuihua L, Kato R, Permata TBM, Iijima M, Yajima H, Yasuhara T, Yamada S, Hasegawa S, Shibata A. RNF8 promotes high linear energy transfer carbon-ion-induced DNA double-stranded break repair in serum-starved human cells. DNA Repair (Amst). 2020; 91–2:102872. 10.1016/j.dnarep.2020.102872 32502756

[34] Wang H, Zhang X, Wang P, Yu X, Essers J, Chen D, Kanaar R, Takeda S, Wang Y. Characteristics of DNA-binding proteins determine the biological sensitivity to high-linear energy transfer radiation. Nucleic Acids Res. 2010; 38:3245–51. 10.1093/nar/gkq069 20150414 PMC2879532

[35] Okayasu R, Okada M, Okabe A, Noguchi M, Takakura K, Takahashi S. Repair of DNA damage induced by accelerated heavy ions in mammalian cells proficient and deficient in the non-homologous end-joining pathway. Radiat Res. 2006; 165:59–67. 10.1667/rr3489.1 16392963

[36] Gerelchuluun A, Manabe E, Ishikawa T, Sun L, Itoh K, Sakae T, Suzuki K, Hirayama R, Asaithamby A, Chen DJ, Tsuboi K. The major DNA repair pathway after both proton and carbon-ion radiation is NHEJ, but the HR pathway is more relevant in carbon ions. Radiat Res. 2015; 183:345–56. 10.1667/RR13904.1 25738894 PMC5684887

[37] Wu X, Yang Z, Liu Y, Zou Y. Preferential localization of hyperphosphorylated replication protein A to double-strand break repair and checkpoint complexes upon DNA damage. Biochem J. 2005; 391:473–80. 10.1042/BJ20050379 15929725 PMC1276948

[38] Loveday BPT, Lipton L, Thomson BN. Pancreatic cancer: An update on diagnosis and management. Aust J Gen Pract. 2019; 48:826–31. 10.31128/AJGP-06-19-4957 31774983

[39] Wang D, Liu R, Zhang Q, Luo H, Chen J, Dong M, Wang Y, Ou Y, Liu Z, Sun S, Yang K, Tian J, Li Z, Wang X. Charged Particle Irradiation for Pancreatic Cancer: A Systematic Review of *In Vitro* Studies. Front Oncol. 2022; 11:775597. 10.3389/fonc.2021.775597 35059313 PMC8764177

[40] Sun X, Wang Y, Ji K, Liu Y, Kong Y, Nie S, Li N, Hao J, Xie Y, Xu C, Du L, Liu Q. NRF2 preserves genomic integrity by facilitating ATR activation and G2 cell cycle arrest. Nucleic Acids Res. 2020; 48:9109–23. 10.1093/nar/gkaa631 32729622 PMC7498319

[41] Laronga C, Yang HY, Neal C, Lee MH. Association of the cyclin-dependent kinases and 14-3-3 sigma negatively regulates cell cycle progression. J Biol Chem. 2000; 275:23106–12. 10.1074/jbc.M905616199 10767298

[42] Fa P, Qiu Z, Wang QE, Yan C, Zhang J. A Novel Role for RNF126 in the Promotion of G2 Arrest via Interaction With 14-3-3σ. Int J Radiat Oncol Biol Phys. 2022; 112:542–53. 10.1016/j.ijrobp.2021.09.025 34563636 PMC8748417

[43] Goodhead DT. Energy deposition stochastics and track structure: what about the target? Radiat Prot Dosimetry. 2006; 122:3–15. 10.1093/rpd/ncl498 17276998

[44] Friedland W, Dingfelder M, Kundrát P, Jacob P. Track structures, DNA targets and radiation effects in the biophysical Monte Carlo simulation code PARTRAC. Mutat Res. 2011; 711:28–40. 10.1016/j.mrfmmm.2011.01.003 21281649

[45] Antonelli F, Campa A, Esposito G, Giardullo P, Belli M, Dini V, Meschini S, Simone G, Sorrentino E, Gerardi S, Cirrone GA, Tabocchini MA. Induction and Repair of DNA DSB as Revealed by H2AX Phosphorylation Foci in Human Fibroblasts Exposed to Low- and High-LET Radiation: Relationship with Early and Delayed Reproductive Cell Death. Radiat Res. 2015; 183:417–31. 10.1667/RR13855.1 25844944

[46] Keta O, Petković V, Cirrone P, Petringa G, Cuttone G, Sakata D, Shin WG, Incerti S, Petrović I, Ristić Fira A. DNA double-strand breaks in cancer cells as a function of proton linear energy transfer and its variation in time. Int J Radiat Biol. 2021; 97:1229–40. 10.1080/09553002.2021.1948140 34187289

[47] Ochs F, Somyajit K, Altmeyer M, Rask MB, Lukas J, Lukas C. 53BP1 fosters fidelity of homology-directed DNA repair. Nat Struct Mol Biol. 2016; 23:714–21. 10.1038/nsmb.3251 27348077

[48] Miller RE, Leary A, Scott CL, Serra V, Lord CJ, Bowtell D, Chang DK, Garsed DW, Jonkers J, Ledermann JA, Nik-Zainal S, Ray-Coquard I, Shah SP, et al. ESMO recommendations on predictive biomarker testing for homologous recombination deficiency and PARP inhibitor benefit in ovarian cancer. Ann Oncol. 2020; 31:1606–22. 10.1016/j.annonc.2020.08.2102 33004253

[49] Andersen SD, Keijzers G, Rampakakis E, Engels K, Luhn P, El-Shemerly M, Nielsen FC, Du Y, May A, Bohr VA, Ferrari S, Zannis-Hadjopoulos M, Fu H, Rasmussen LJ. 14-3-3 checkpoint regulatory proteins interact specifically with DNA repair protein human exonuclease 1 (hEXO1) via a semi-conserved motif. DNA Repair (Amst). 2012; 11:267–77. 10.1016/j.dnarep.2011.11.007 22222486 PMC4586177

[50] Engels K, Giannattasio M, Muzi-Falconi M, Lopes M, Ferrari S. 14-3-3 Proteins regulate exonuclease 1-dependent processing of stalled replication forks. PLoS Genet. 2011; 7:e1001367. 10.1371/journal.pgen.1001367 21533173 PMC3077382

[51] Tomimatsu N, Mukherjee B, Harris JL, Boffo FL, Hardebeck MC, Potts PR, Khanna KK, Burma S. DNA-damage-induced degradation of EXO1 exonuclease limits DNA end resection to ensure accurate DNA repair. J Biol Chem. 2017; 292:10779–90. 10.1074/jbc.M116.772475 28515316 PMC5491765

[52] Matsuoka S, Ballif BA, Smogorzewska A, McDonald ER 3rd, Hurov KE, Luo J, Bakalarski CE, Zhao Z, Solimini N, Lerenthal Y, Shiloh Y, Gygi SP, Elledge SJ. ATM and ATR substrate analysis reveals extensive protein networks responsive to DNA damage. Science. 2007; 316:1160–6. 10.1126/science.1140321 17525332

[53] Xu X, Vaithiyalingam S, Glick GG, Mordes DA, Chazin WJ, Cortez D. The basic cleft of RPA70N binds multiple checkpoint proteins, including RAD9, to regulate ATR signaling. Mol Cell Biol. 2008; 28:7345–53. 10.1128/MCB.01079-08 18936170 PMC2593429

[54] Lukas C, Falck J, Bartkova J, Bartek J, Lukas J. Distinct spatiotemporal dynamics of mammalian checkpoint regulators induced by DNA damage. Nat Cell Biol. 2003; 5:255–60. 10.1038/ncb945 12598907

[55] Bahassi EM, Ovesen JL, Riesenberg AL, Bernstein WZ, Hasty PE, Stambrook PJ. The checkpoint kinases Chk1 and Chk2 regulate the functional associations between hBRCA2 and Rad51 in response to DNA damage. Oncogene. 2008; 27:3977–85. 10.1038/onc.2008.17 18317453

[56] Tian H, Faje AT, Lee SL, Jorgensen TJ. Radiation-induced phosphorylation of Chk1 at S345 is associated with p53-dependent cell cycle arrest pathways. Neoplasia. 2002; 4:171–80. 10.1038/sj.neo.7900219 11896572 PMC1550321

[57] Lai KK, Chan KT, Choi MY, Wang HK, Fung EY, Lam HY, Tan W, Tung LN, Tong DK, Sun RW, Lee NP, Law S. 14-3-3σ confers cisplatin resistance in esophageal squamous cell carcinoma cells via regulating DNA repair molecules. Tumour Biol. 2016; 37:2127–36. 10.1007/s13277-015-4018-6 26346170

[58] Shen W, Song Z, Zhong X, Huang M, Shen D, Gao P, Qian X, Wang M, He X, Wang T, Li S, Song X. Sangerbox: A comprehensive, interaction-friendly clinical bioinformatics analysis platform. iMeta. 2022; 1:e36. 10.1002/imt2.36 38868713 PMC10989974

[59] Blum A, Wang P, Zenklusen JC. SnapShot: TCGA-Analyzed Tumors. Cell. 2018; 173:530. 10.1016/j.cell.2018.03.059 29625059

