# Supplementary material for: 14-3-3σ downregulation sensitizes pancreatic cancer to carbon ions by suppressing the homologous recombination repair pathway
Source: Aging (Albany NY). 2024 Jun 5;16(11):9727–52. doi: 10.18632/aging.205896 (PMC11210243; doi:10.18632/aging.205896)
Supplement: Supplementary Figures [file aging-16-205896-s001.pdf]

SUPPLEMENTARY FIGURES

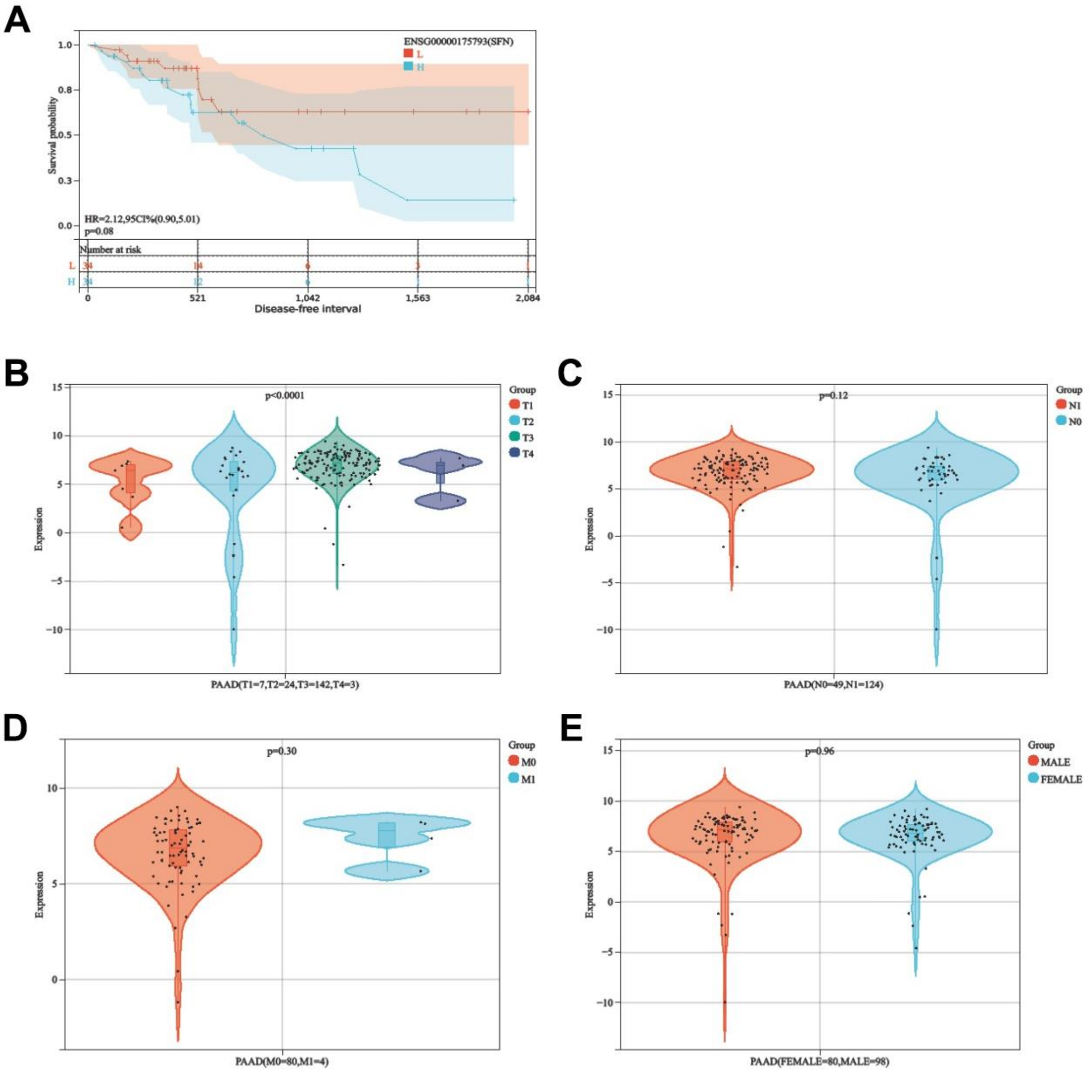

Supplementary Figure 1. (**A**) The K-M survival curves are plotted to predict the DFS of TCGA patients. (**B**–**E**) The expression of 14-3-3σ in PAAD tissues in patients with different T (**B**), N (**C**), M (**D**) stages, and sex (**E**).

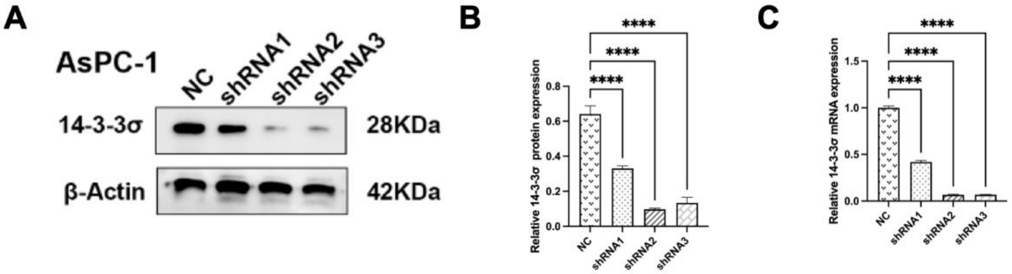

Supplementary Figure 2. (**A**) The protein and mRNA levels of 14-3-3σ in AsPC-1 cells with 14-3-3σ-shRNA were detected by immunoblotting (**A**, **B**) and qPCR (**C**).
