# Supplementary material for: 14-3-3σ downregulation sensitizes pancreatic cancer to carbon ions by suppressing the homologous recombination repair pathway
Source: Aging (Albany NY). 2024 Jun 5;16(11):9727–52. doi: 10.18632/aging.205896 (PMC11210243; doi:10.18632/aging.205896)
Supplement: Supplementary Tables [file aging-16-205896-s002.pdf]

SUPPLEMENTARY TABLES

Supplementary Table 1. The original data of surviving fraction of AsPC-1 cells.

Dose	Carbon ions		si14-3-3σ + Carbon ions	
	Mean	SD	Mean	SD
0 Gy	1.000	0.120	1.000	0.134
1 Gy	0.546	0.068	0.363	0.086
2 Gy	0.170	0.031	0.072	0.019
3 Gy	0.028	0.003	0.010	0.004

Abbreviation: SD, Standard Deviation.

Supplementary Table 2. The original data of surviving fraction of MiaPaCa-2 cells.

Dose	Carbon ions		14-3-3σ + Carbon ions	
	Mean	SD	Mean	SD
0 Gy	1.000	0.053	1.000	0.065
1 Gy	0.201	0.027	0.350	0.035
2 Gy	0.039	0.007	0.100	0.016
3 Gy	0.008	0.001	0.021	0.004

Abbreviation: SD, Standard Deviation.

Supplementary Table 3. Sequence information used in this study.

ID	Sequences or target sequence (5’→3’)
14-3-3σ si 1	GUCCAGUAUUGAGCAGAAATTUUUCUGCUCAAUACUGGACTT
14-3-3σ si 2	GGGUCUUCUACCUGAAGAUTTAUCUUCAGGUAGAAGACCCTT
14-3-3σ si 3	CCAUGGACACAUCAGCAAGAATTUUCUUGCUGAUGUCCAUGGTT
Negative control siRNA	UUCUCCGAACGUGUCACGUTTACGUGACACGUUCGGAGAATT
14-3-3σ shRNA	ACGACAAGAAGCGCATCATTG
Lentivirus NC	TTCTCCGAACGTGTCACGT
14-3-3σ OE sequence	ATGGAGAGAGCCAGTCTGATCCAGAAGGCCAAGCTGGCAGAGCAGGCC<br>GAACGCTATGAGGACATGGCAGCCTTCATGAAAGGCGCCGTGGAGAAG<br>GGCGAGGAGCTCTCCTGCGAAGAGCGAAACCTGCTCTCAGTAGCCTATA<br>AGAACGTGGTGGGCGGCCAGAGGGCTGCCTGGAGGGTGCTGTCCAGTA<br>TTGAGCAGAAAAGCAACGAGGAGGGCTCGGAGGAGAAGGGGCCCGAG<br>GTGCGTGAGTACCGGGAGAAGGTGGAGACTGAGCTCCAGGGCGTGTGC<br>GACACCGTGCTGGGCCTGCTGGACAGCCACCTCATCAAGGAGGCCGGG<br>GACGCCGAGAGCCGGGTCTTCTACCTGAAGATGAAGGGTGACTACTAC<br>CGCTACCTGGCCGAGGTGGCCACCGGTGACGACAAGAAGCGCATCATT<br>GACTCAGCCCGGTCAGCCTACCAGGAGGCCATGGACATCAGCAAGAAG<br>GAGATGCCGCCCACCAACCCCATCCGCCTGGGCCTGGCCCTGAACTTTT<br>CCGTCTTCCACTACGAGATCGCCAACAGCCCCGAGGAGGCCATCTCTCT<br>GGCCAAGACCACTTTCGACGAGGCCATGGCTGATCTGCACACCCTCAGC<br>GAGGACTCCTACAAAGACAGCACCCTCATCATGCAGCTGCTGCGAGAC<br>AACCTGACACTGTGGACGGCCGACAACGCCGGGGAAGAGGGGGGCGA<br>GGCTCCCCAGGAGCCCCAGAGCTGA

Supplementary Table 4. Antibodies used in this study.

Antibodies	Application	Company	Catalog numbers
14-3-3σ	1:5000 for WB	Proteintech	66251-1-Ig
p-CHK1	1:2000 for WB	Immunoway	YP0063
CHK1	1:2000 for WB	Immunoway	YT0902
RPA2	1:2000 for WB	Immunoway	YT4170
RPA2	1:200 for IHC	Immunoway	YT4170
RPA2	1:2000 for IF	Proteintech	67999-1-Ig
p-RPA2	1:2000 for WB	Immunoway	YP0476
p-CHK2	1:2000 for WB	Immunoway	YP0065
CHK2	1:2000 for WB	Immunoway	YT0908
p-ATM	1:2000 for WB	Immunoway	YP1206
ATM	1:2000 for WB	Immunoway	YT0397
p-ATR	1:2000 for WB	Immunoway	YP1269
ATR	1:2000 for WB	Immunoway	YT0416
γ H2AX	1:2000 for WB	Cell Signaling Technology	9718
γ H2AX	1:200 for IF	Cell Signaling Technology	9718
γ H2AX	1:200 for IHC	Cell Signaling Technology	9718
RAD51	1:3000 for WB	GeneTex	GTX100469
RAD51	1:200 for IF	GeneTex	GTX100469
53BP1	1:3000 for WB	GeneTex	GTX102595
53BP1	1:200 for IF	GeneTex	GTX102595
Ki67	1:400 for IHC	Proteintech	28074-1-AP
β-Actin	1:2000 for WB	Proteintech	66009-1-Ig

Supplementary Table 5. Primer sequences used for amplification

Gene	Sequences or target sequence (5'→3')
14-3-3σ Fp	GGATCCCACTCTTCTTGCAG
14-3-3σ Rp	CTGTCCAGTTCTCAGCCACA
β-actin Fp	CGGAACCGCTCATTGCC
β-actin Rp	ACCCACACTGTGCCCATCTA
ATM Fp	TGCACTTCCGTCAGCAAAGA
ATM Rp	GCGCTTACACATCTCTTCCAC
ATR Fp	CGTGATCAGCGAGAGCCTTT
ATR Rp	ACATGGGTCTTGGCCTTTTCA
